# Analyzing pharmacological intervention points: A method to calculate external stimuli to switch between steady states in regulatory networks

**DOI:** 10.1371/journal.pcbi.1007075

**Published:** 2019-07-16

**Authors:** Tim Breitenbach, Chunguang Liang, Niklas Beyersdorf, Thomas Dandekar

**Affiliations:** 1 Lehrstuhl für Bioinformatik, Biozentrum, Universität Würzburg, Würzburg, Germany; 2 Institut für Virologie und Immunbiologie, Universität Würzburg, Würzburg, Germany; University of Virginia, UNITED STATES

## Abstract

Once biological systems are modeled by regulatory networks, the next step is to include external stimuli, which model the experimental possibilities to affect the activity level of certain network’s nodes, in a mathematical framework. Then, this framework can be interpreted as a mathematical optimal control framework such that optimization algorithms can be used to determine external stimuli which cause a desired switch from an initial state of the network to another final state. These external stimuli are the intervention points for the corresponding biological experiment to obtain the desired outcome of the considered experiment. In this work, the model of regulatory networks is extended to controlled regulatory networks. For this purpose, external stimuli are considered which can affect the activity of the network’s nodes by activation or inhibition. A method is presented how to calculate a selection of external stimuli which causes a switch between two different steady states of a regulatory network. A software solution based on Jimena and Mathworks Matlab is provided. Furthermore, numerical examples are presented to demonstrate application and scope of the software on networks of 4 nodes, 11 nodes and 36 nodes. Moreover, we analyze the aggregation of platelets and the behavior of a basic T-helper cell protein-protein interaction network and its maturation towards Th0, Th1, Th2, Th17 and Treg cells in accordance with experimental data.

## Author summary

Organisms can be seen as molecular networks being able to react on external stimuli. Experiments are performed to understand the underlying regulating mechanisms within the molecular network. A common purpose for these efforts is to reveal mechanisms with which the molecular networks can be affected to achieve a desired behavior. To cover the complexity of life these models of molecular networks often need to be quite huge and need to have many cross connections between the different agents of the network that regulate the behavior of the network. A useful tool to structure this complexity are mathematical methods. Once the model based on experiments is set up the experimental data can be further processed by mathematical methods. As experiments are cumbersome, the present work provides a framework that can be used to systematically figure out intervention points in molecular networks to cause a desired effect. In this way promising intervention strategies can be obtained. For instance the process of obtaining new drugs for pharmacological modulation can be shortened as in the best case the calculated intervention strategy just has to be validated with one experiment and the time consuming procedure of searching an intervention strategy with several experiments can be saved.

## Introduction

Biological networks are often formed by interacting proteins and molecules. Their change in time is often biologically regulated to adapt to different conditions. Detailed mathematical models describe changes of proteins, for instance their phosphorylation state or activity in general by differential equations. We analyze here in particular the question, how we can calculate the result of a pharmacological intervention and identify the best target points (receptors or downstream in the cascade or any other point in the network) to shift the network state into a new activity pattern as desired (either for medical or for research purpose). Our approach thus allows the user to define the intervention points of choice and we then systematically calculate the optimal steering options the user has available to drive the network then into the desired state. We include the information how well the objective can be met with this choice.

Mathematically speaking, regulatory networks are commonly modeled by a system of coupled ordinary differential equations (ODEs) [1]. Then, a network is analyzed with respect to its steady states of the corresponding system of coupled ODEs [2, 3] and associated with stable states observed in the corresponding real network like an expression pattern of different genes influencing each other in a cell. If once the system is in a steady state, it remains there for all times and it is not able to switch its state. Such an approach considers the network as a closed system where there are no interactions with the environment, in contrast to an open system which interacts with its environment. Usually, one models just a section of reality. This section has to interact with its environment such that we can notice it. Out of this thought, we realize that a model is supposed to consider interactions with the environment in which it is embedded. Following this concept, we extend the model presented in [3] and [1] by external stimuli. These external stimuli act on certain nodes of the network by increasing or decreasing the level of activation of a node, called activation or inhibition, respectively. A catchy example for including external stimuli into regulatory networks is signalling between different cells. For instance the secretion of interferons or interleukins secreted by T-cells can serve as an external stimulus for another cell type of the immune system. Further, based on the concept of regulatory networks with external stimuli, one can couple different model networks to see if the steady state of one network where some certain nodes are active can induce a switch in another model network where these active nodes of the first network affect the second network as external stimuli.

In fact, we show in the present work that these external stimuli can cause a switch between an initial steady state of a network and a desired final steady state of the network. That means that we address the observation that a real system can change its quite stable state if an external perturbation is strong enough to cause this switch. A plausible example is the differentiation of stem cells. Different tissues are associated with different steady states of the corresponding regulatory network. Now, by applying agents which are the mentioned external stimuli, the stem cells differentiate to different tissues, corresponding to the external stimuli, see for example [4, 5]. Another issue is that the cells can even be reprogrammed that means that the cell being a certain type of tissue changes its type of tissue, see for example [6]. A further special case is the switch from a cancer cell to apoptosis [7, 8] where each genetic expression program associated with a cancer cell or apoptosis, respectively, can be associated with a certain steady state of an appropriate network of ODEs.

Our approach provides a rational method to analyze networks with respect to the influence of external stimuli on the considered network, especially its steady states. For this purpose, for example Jimena [3], SQUAD [2] or [1] aim at analyzing and finding steady states of a network where no external stimulus is applied to. This method and software tool presented in the present work is a systematical approach to steady state analysis and the transfer of one steady system state to a different one. This can be achieved by interpreting the external stimuli framework as an optimal control framework among others and thus different algorithms can be used in order to determine appropriate external stimuli for the desired switch. With the virtue of this framework it is possible to analyze huge networks (more than 100 nodes) where simultaneously possible intervention points, which are the external stimuli and can be drugs for instance, are registered, that can even affect several nodes at once, that means the expression of several genes at once. Then one can calculate the best drug combination that influences the network in the desired direction from all the information of interaction and intervention points that is encoded in the interaction graph of the nodes with the corresponding external stimuli.

In Subsection “An extension of regulatory networks with external stimuli” (Methods), we give a mathematical description of the model presented in [1] and explain in detail how to model these external stimuli by mathematical equations and how to extend the mathematical model by external stimuli, followed by Subsection “A mathematical calculus to determine external stimuli”, the mathematical calculus to determine external stimuli causing the desired switch between two different steady states of the network. For this purpose, the problem is formulated as an optimal control problem where a target functional is minimized subject to constraints. The corresponding optimum of the target functional is obtained by the optimal controls that correspond to optimal external stimuli. The optimal controls are calculated essentially by a Lagrange ansatz or alternatively by the Pontryagin maximum principle. In order to solve the resulting equations, a projected gradient method and a sequential quadratic Hamiltonian method are implemented. In Subsection “Analysis of the aggregation of platelets”, we analyze a regulatory network for platelet aggregation proposed in [9] that is fitted to experimental data. We validate our framework by finding these external stimuli that trigger aggregation in the corresponding experiment. In Subsection “A switch between two different types of T-helper cells”, we investigate the model for T-cell differentiation into Th1, Th2, Th17 or Treg from Th0 proposed in [10], apply the framework of external stimuli and predict strategies how to switch from the Th17 cell type to the Treg cell type. Furthermore, we provide a discussion of these results to validate our method in a biological context. For this purpose, we compare our calculated interventions with experimental evidence from three experimental groups confirming our interventions for the calculated switch from Th17 cell type to Treg cell type.

In the Discussion we compare our software package to alternative software dealing with the analysis of regulatory networks and explain how to combine different software packages to bunch it together to a great tool for network analysis and modeling with regulatory networks. Furthermore we discuss the sensitivity of the proposed method and how the method can be used for modeling taking systematically the external stimuli into account. We conclude the Discussion by illustrating the realization of our predictions. A conclusion completes the Discussion. Furthermore, besides the Matlab codes, supplementary material is provided with the present work where mathematical and algorithmic details of the used methods are explained.

## Materials and methods

Here, we describe the basic ingredients of our framework. In the S1 File there is a detailed description of the underlying mathematical calculations and utilized algorithms. Furthermore, S1 File presents all figures as they are obtained directly from the Matlab scripts so that the reader knows how the output from the scripts will look like. In the results section of the paper, however, we polished the output further (proper axis labeling according to biological entity and biological interpretation).

### An extension of regulatory networks with external stimuli

**Fig 1:**
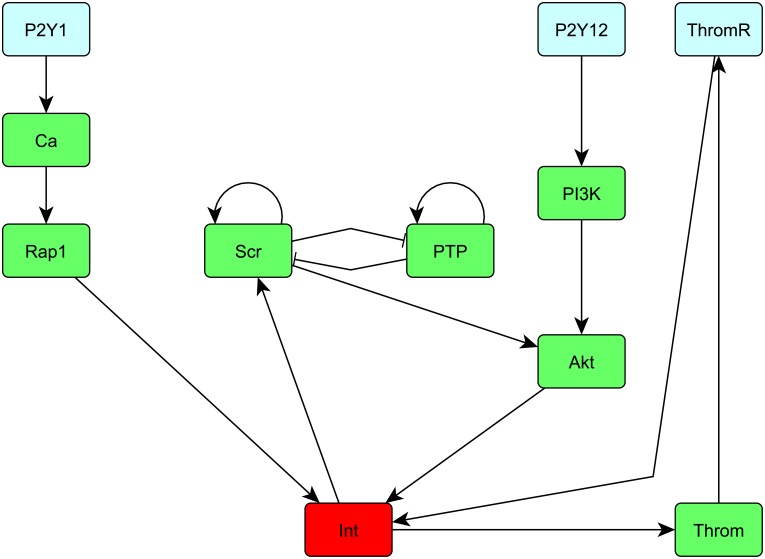
Schematic of the platelet network from [9, Figure 1].

An extension of regulatory networks with external stimuli hence means that we can mathematically describe the result of network interventions, either pharmacological or by molecular manipulations such as RNAi, knock downs or any protein inactivation by other methods including chemical or genetic activity inhibition in the following only mentioned as “knock down” as well as any other method, e.g. changing environmental conditions such as pH. In this subsection, we extend the model for continuous regulatory networks described in [1] such that our model includes external stimuli. Their model is a set of equations that can be used to translate the graph of any regulatory network into a continuous dynamical system. Hence these are all differential equations as shown in detail in the following, see Eq (1). Following (1), we insert the activating and inhibiting nodes of the considered networks. By our provided software only the graphs depicted in the corresponding figure in this work are needed to create the corresponding equations. If there is a need to see the equations used for the calculations here or in results, please see the provided Matlab files where the equations are implemented in the main-file that are available for downloading. Our model extends this now by ordinary differential equations for external stimuli, see (2). Eq (2) can also be obtained with the provided software by inputting the graph depicted in Fig 1. By these external stimuli, we can influence the network, which may result in a switch between different steady states of the regulatory network. We start with a network of nodes where the change in time of the activation level $x_{k}:R_{0}^{+}\to [0,1]$ of each node *k* ∈ {1, …, *n*} is described by the following ordinary differential equation

$$\begin{array}{c} \frac{dx_{k}}{dt}=\frac{-e^{\frac{1}{2}h}+e^{-h(\omega_{k}-\frac{1}{2})}}{\left(1-e^{\frac{1}{2}h})(1+e^{-h(\omega_{k}-\frac{1}{2})}\right)}-\gamma_{k}x_{k} \end{array}$$ (1)

with

$$\begin{array}{c} \omega_{k}=\{\begin{array}{cc} AI & \text{if}\;x_{k}\;\text{has}\;\text{activators}\;\text{and}\;\text{inhibitors}\\ A & \text{if}\;x_{k}\;\text{has}\;\text{only}\;\text{activators}\\ I & \text{if}\;x_{k}\;\text{has}\;\text{only}\;\text{inhibitors} \end{array} \end{array}$$

and

$$\begin{array}{c} A=(\frac{1+\sum_{j\in A_{k}}\alpha_{j}^{k}}{\sum_{j\in A_{k}}\alpha_{j}^{k}})(\frac{\sum_{j\in A_{k}}\alpha_{j}^{k}x_{j}}{1+\sum_{j\in A_{k}}\alpha_{j}^{k}x_{j}}),\\ I=1-(\frac{1+\sum_{j\in I_{k}}\beta_{j}^{k}}{\sum_{j\in I_{k}}\beta_{j}^{k}})(\frac{\sum_{j\in I_{k}}\beta_{j}^{k}x_{j}}{1+\sum_{j\in I_{k}}\beta_{j}^{k}x_{j}}) \end{array}$$

where the activators of node *k* are elements of the subset {*x*_*j*_| *j* ∈ *A*_*k*_ ⊆ {1, …, *n*}} ⊆ {*x*_*k*_| *k* ∈ {1, …, *n*}} where *A*_*k*_ contains all the indices of {1, …, *n*} of the nodes which activate node *k* and the corresponding $\alpha_{j}^{k}>0$ weights the contribution of the activation level *x*_*j*_ of node *j* to the total activation of node *k*. Analogously, the inhibitors of node *k* are elements of the subset {*x*_*j*_| *j* ∈ *I*_*k*_ ⊆ {1, …, *n*}} ⊆ {*x*_*k*_| *k* ∈ {1, …, *n*}} where *I*_*k*_ contains all the indices of {1, …, *n*} of the nodes which inhibit node *k* and the corresponding $\beta_{j}^{k}>0$ weights the contribution of the activation level *x*_*j*_ of node *j* to the total inhibition of node *k*. Furthermore, *h* > 0 where *h* models the cooperativity. If *h* is big, then the behavior of the equation is close to a switcher while small *h* are closer to a linear behavior of the activation level with respect to the input activation level. According to [1], the first term of (1) is called the activation function or activation term and the second term is called the decay where *γ*_*k*_ > 0 models the speed of decay of the activation level *x*_*k*_ of node *k*.

In the next step, we show how to extend models consisting of ordinary differential equations with external stimuli on the example of the model (1). External stimuli are all the intervention possibilities that an experimenter has to influence the considered system or the modeled experiment, respectively. For this purpose, we define the set of all external stimuli *S* ≔ {*u*_*j*_| *j* ∈ {1, …, *m*}} where $u_{j}:R_{0}^{+}\to [0,1]$. In our model *u*_*j*_ = 0 means that the external stimulus is not active while the activity level of the stimulus is supposed to be linearly interpolated to its maximum activity level that is modeled by *u*_*j*_ = 1. An activation of node *k* by an external stimulus *j* is modeled by adding *σ*_*kj*_ *u*_*j*_ (1 − *x*_*k*_) to the right hand-side of (1). That means if the external stimulus *u*_*j*_ ≡ 0, then there is no activation of node *k* by the external stimulus *j* and if node *k* has full activity, then the external stimulus has also no effect on the activation level *x*_*k*_ of node *k*. Analogously, an inhibition of node *k* by an external stimulus *j* is modeled by subtracting *η*_*kj*_*u*_*j*_*x*_*k*_ from the right hand-side of (1). That means, if the external stimulus *u*_*j*_ ≡ 0, then there is no inhibition of node *k* by the external stimulus *j* and if node *k* has no activity, then the external stimulus *j* has no effect on the activation level *x*_*k*_ of node *k*. The parameters *σ*_*kj*_ ≥ 0 and *η*_*kj*_ ≥ 0 are used to fit the value *u*_*j*_ to experimental activation or inhibition of nodes caused by the modeled external stimulus or can be used to weight the contribution of external stimulus *u*_*j*_ to the activation or inhibition, respectively, of node *k* where *σ*_*kj*_ = 0 or *η*_*kj*_ = 0 means that external stimulus *j* does not directly effect node *k*. Our model for the change in time of the activation level *x*_*k*_ of node *k* is given as in the discussion above but exchanging (1) by

$$\begin{array}{c} \frac{dx_{k}}{dt}=\frac{-e^{\frac{1}{2}h}+e^{-h(\omega_{k}-\frac{1}{2})}}{\left(1-e^{\frac{1}{2}h})(1+e^{-h(\omega_{k}-\frac{1}{2})}\right)}-\gamma_{k}x_{k}+\sum_{j=1}^{m}\sigma_{kj}u_{j}(1-x_{k})-\sum_{j=1}^{m}\eta_{kj}u_{j}x_{k} \end{array}$$ (2)

for *k* ∈ {1, …, *n*}.

We remark that in the framework of modeling, without any external stimuli, we have an unperturbed network. That especially means that the values of the steady states of a regulatory network are independent of the external stimuli. Thus these steady states are a priori given or computable, respectively, without knowing what external stimuli might effect the regulatory network. This is reasonable because if the steady states of regulatory network model all the genetic programs which a cell can perform for instance, then these programs are an intrinsic property of this cell independent of possible external stimuli which ever can exist. However, an external stimulus might cause a change from one genetic program to another if applied.

Next, we illustrate how these external stimuli can be realized in a real experiment. In general, we say that the activation level of a node is high if the product which corresponds to the node has a high concentration and is low if the corresponding product’s concentration is low. An example is the expression of a protein by a gene. If the concentration of the protein is high, then we say that the corresponding gene is at a high activation level and analogously reverse. That means, that activation of a node is every operation on the node which increases the concentration of the product associated with a certain node. Therefore, increasing the activation level can be done by adding the product of the corresponding node to the system with which it influences other nodes, like RNA or protein. This simulates a higher activation level of the corresponding node. Another effect which increases the activity of the node is adding a substrate to the system which improves transcription or translation. Imaginable is the activation of an enhancer region close to a promoter associated to a node. We emphasize that these effects are not like a knock in of a gene as this operation changes the topology of the network which means that nodes or edges are added to the network.

The inhibition of a node in model (2) means that the concentration of the product associated with that node is decreased, like an additional decay. This can be done, for example, by antibodies which bind to the product, degradation of the product by enzymes or any other modification, like post transcriptional or post translational modifications, at the product which inhibits its intended function in the system and thus converts the product to a biologically inactive form. Consequently the concentration of biologically active product decreases and this is considered as a decay of the activation level of the corresponding node. This is associated with a so called knock down. Analogously to a knock in, a knock out changes the topology of the network as well and corresponds to the deletion of a node or an edge from the network. This operation is not modeled by the action of the external stimuli within the framework proposed in this work.

Model (2) can be extended to external stimuli which act on the transcriptional and translational level in the following way. Maybe a substrate cannot decrease the concentration of the product *x*_*k*_ of a corresponding node *k* but its activation term. For example, if one blocks areas in the promoter region by oligopeptides such that transcription factors can bind worse to the DNA, then the expression of the corresponding product slows down as the activation term is smaller. Such a model can be formulated as follows

$$\begin{array}{c} \begin{array}{cc} \frac{dx_{k}}{dt} & =\frac{-e^{\frac{1}{2}h}+e^{-h(\omega_{k}-\frac{1}{2})}}{\left(1-e^{\frac{1}{2}h})(1+e^{-h(\omega_{k}-\frac{1}{2})}\right)}(\prod_{j=1}^{m}(1-\zeta_{kj}u_{j}))-\gamma_{k}x_{k}\\ & +\sum_{j=1}^{m}\sigma_{kj}u_{j}(1-x_{k})-\sum_{j=1}^{m}\eta_{kj}u_{j}x_{k} \end{array} \end{array}$$ (3)

where 0 ≤ *ζ*_*kj*_ ≤ 1. If *ζ*_*kj*_ = 0 for all *k* and *j*, then model (3) transforms into (2). By the coefficients *ζ*_*kj*_, one can adjust how much the external stimulus *j* affects the activation term of the node *k* even at full activity of *u*_*j*_. That means that for *ζ*_*kj*_ = 0, the external stimulus *j* has no effect on node *k* and for *ζ*_*kj*_ = 1, a fully activated external stimulus *j*, that means *u*_*j*_ = 1, totally prevents the expression of the product of node *k*. If 0 < *ζ*_*kj*_ < 1, then even a fully activated external stimulus *j* cannot totally prevent the expression. This captures the nature of an equilibrium reaction as it appears when, for example, transcription factors compete with other substrates in binding to the DNA. Therefore, *ζ*_*kj*_ can also be used to fit the influence of *u*_*j*_ to experimental data.

Besides drugs or some other chemicals acting as external stimuli, there are further external stimuli like physical signals. For example light or mechanical stress, which is detected by receptors and is converted into activation or inhibition of a node. For instance, DNA damage caused for example by X-rays activates p53 [11] or temperature sensed by RNA thermometers can chance expression patterns [12]. This can be modeled within our framework by covering the effects of these external stimuli by the functions *u*_*j*_, *j* ∈ {1, …, *m*} that activate or inactivate the corresponding nodes. We remark that this is a very effective modeling as we just consider the essential effects of the interactions within a real system that we model.

In order to compare the results from these models with results from an experiment in detail, one has to check if the behavior of the real network is the same like the one of the model network when applying an external stimulus as far as the nodes’ activity is concerned. Furthermore, one can check if an external stimulus has the same effect on a node’s activity like used in the model in order to adjust the coefficients *σ*_*kj*_, *η*_*kj*_ and *ζ*_*kj*_. In this way, the coupling strength of an external stimulus can be taken into account. For example, if an inhibiting external stimulus *j* supposed to cause a knock down of node *k* cannot force a node’s activation level below a certain level although fully applied, then one can adjust the corresponding coefficient *η*_*kj*_ or *ζ*_*kj*_ until the model has the same behavior. Analogously, if an external stimulus cannot steer a node’s activation level to its full amplitude although fully applied, then one can adjust the corresponding coefficient *σ*_*kj*_ until the model shows the same behavior like the real system. One should also check if the external stimulus like a certain chemical agent used to put the external stimulus from the model into effect has an exclusive effect on the corresponding node in the real system or if the utilized agent has an multi target effect on several nodes in the real system. If the utilized external stimulus has multi target effects on several nodes, then such an stimulus *j* can be considered with the model above in that way that the coefficients *ζ*_*kj*_, *σ*_*kj*_ or *η*_*kj*_ are greater then zero for more than just one *k*. Then the external stimulus *j* appears in more then one equation meaning that it has an effect on the corresponding nodes.

### A mathematical calculus to determine external stimuli

In this mathematical part we discuss how to formulate the task of calculating external stimuli that shift the regulatory network into a new state such that a given set of nodes are modulated and a given network state is achieved as a mathematical optimal control problem. Specifically, we present a way how to calculate external stimuli which are able to switch the regulatory network from one steady state to another.

Let $x_{0}≔(\begin{array}{c} x_{1}^{0}\\ \vdots \\ x_{n}^{0} \end{array})\in R^{n}$ denote the steady state in which our regulatory network starts and let $x_{d}≔(\begin{array}{c} x_{1}^{d}\\ \vdots \\ x_{n}^{d} \end{array})\in R^{n}$ denote the steady state in which we desire our regulatory network to be. We consider *x*_*d*_ as a constant function over time. The activation levels *x*_*k*_ follow the dynamics given by (2) for *t* ∈ (0, *T*) where we have *T* > 0 and $x_{k}(0)=x_{k}^{0}$ for all *k* ∈ {1, …, *n*}. Next, we formulate the distance of two steady states with a mathematical function as follows. The smaller the sum of integrals

$$\begin{array}{c} \frac{1}{2}\sum_{k=1}^{n}\int_{0}^{T}{\left|x_{k}(t)-x_{k}^{d}\right|}^{2}dt \end{array}$$ (4)

is, the faster each activation level *x*_*k*_ reaches its desired steady state $x_{k}^{d}$ through the action of our external stimuli. Analogously, the smaller the sum of integrals

$$\begin{array}{c} \sum_{j=1}^{m}\int_{0}^{T}u_{j}(t)dt \end{array}$$ (5)

is, the less external stimuli are needed for switching the steady states, both the number of different external stimuli and the time they are applied to the network. By construction, without any external stimuli, the regulatory network rests in the steady state *x*_0_. If we multiply (5) by *α* > 0 and add it to (4), we obtain

$$\begin{array}{c} J(x,u)≔\frac{1}{2}\sum_{k=1}^{n}\int_{0}^{T}{\left|x_{k}(t)-x_{k}^{d}\right|}^{2}dt+\alpha \sum_{j=1}^{m}\int_{0}^{T}u_{j}(t)dt \end{array}$$ (6)

where $x≔(\begin{array}{c} x_{1}\\ \vdots \\ x_{n} \end{array})$ and $u≔(\begin{array}{c} u_{1}\\ \vdots \\ u_{m} \end{array})$. Minimizing (6) means to bring the regulatory network from the initial point of rest *x*_0_ as close to the desired state *x*_*d*_ as possible, in the best case to the desired steady state, while using as few external stimuli as possible.

The constant *α* weights which term of (6) is more important to be little. If *α* is big, then it is more important to use few external stimuli than to steer the regulatory network to *x*_*d*_. However, it can happen, when *α* is too big, that the external stimuli are calculated as constant zero functions for all times and the regulatory network remains in its initial steady state because its too costly to have non-zero external stimuli which have the desired perturbation on the network. On the other hand, little *α* makes it more important that the regulatory network is steered from *x*_0_ to *x*_*d*_ and the number of non-zero external stimuli might increase as well. Therefore with the constant *α* we can reduce the number of active external stimuli which is important for pharmacological applications. More specific, the constant *α* can be utilized to determine only a small number of efficient external stimuli whose input gives the biggest gain. This is demonstrated in the Supplemental material S1 File, experiment with the Eq. (14).

Roughly spoken, the larger *T* is and the smaller *α* is, the more it is important, that we obtain external stimuli which steer the regulatory network from the initial steady state to the desired one, according to (6). Finally, the mathematical formulation of the problem above is as follows.

Minimize (6) such that $\frac{d}{dt}x_{k}=f_{k}(x,u)$, $x_{k}(0)=x_{k}^{0}$ is fulfilled for all *k* ∈ {1, …, *n*} and *t* ∈ (0, *T*) where *f*_*k*_ (*x*, *u*) is the right hand-side of (2), that means

$$\begin{array}{c} f_{k}(x,u)≔\frac{-e^{\frac{1}{2}h}+e^{-h(\omega_{k}-\frac{1}{2})}}{\left(1-e^{\frac{1}{2}h})(1+e^{-h(\omega_{k}-\frac{1}{2})}\right)}-\gamma_{k}x_{k}+\sum_{j=1}^{m}\sigma_{kj}u_{j}(1-x_{k})-\sum_{j=1}^{m}\eta_{kj}u_{j}x_{k}. \end{array}$$ (7)

This can be equivalently formulated as follows

$$\begin{array}{c} \begin{array}{cc} & \min_{x,u}J(x,u)\\ & s.t.\;\frac{d}{dt}x_{k}=f_{k}(x,u),x_{k}(0)=x_{k}^{0}\;\text{for}\;\text{all}\;k\in \{1,...,n\}\;\text{and}\;t\in (0,T) \end{array} \end{array}$$ (8)

which is called optimal control problem. Solution strategies for solving (8) with respect to finding effective intervention points in regulatory networks can be found in the Supplementary material S File. There we also find the following three algorithms that are used in the present work.

1. Algorithm 1: Projected gradient method
2. Algorithm 2: Sequential quadratic Hamiltonian method
3. Algorithm 3: Combinatorial method

Further in the Supplementary material S1 File we demonstrate the basic application of the optimal control framework (8) to external stimuli analysis. For this purpose, we use a small network of 4 nodes and the dynamics (2) to get familiar with the approach for network analysis and its scope in general before we focus on its application in Subsection “Analysis of the aggregation of platelets” and Subsection “A switch between two different types of T-helper cells” to models with real data in order to validate the framework. We remark that the proposed optimal control framework holds for any dynamics consisting of ordinary differential equations. For example the framework is applicable to dynamics where the term $\frac{-e^{\frac{1}{2}h}+e^{-h(\omega_{k}-\frac{1}{2})}}{\left(1-e^{\frac{1}{2}h})(1+e^{-h(\omega_{k}-\frac{1}{2})}\right)}-\gamma_{k}x_{k}$ in (7) is replaced by terms that are derived from the chemical reaction network theory [13–15]. In general any *f*_*k*_ can be given by any function that defines a well posed right hand-side of an ordinary differential equation.

We conclude this subsection with the following remark about the desired state *x*_*d*_.

*Remark 1* In this framework, the desired state *x*_*d*_ does not need to be a constant function a steady state. It can be a time-dependent function as well. With a time-dependent function one can give a desired trajectory to which the state *x* is supposed to be as close as possible subject to the constraints (2) and to the given set of controls or external stimuli, respectively. For example *x*_*d*_ can model a time-dependent expression pattern where the values for each node vary with respect to time. Moreover, one can think of pharmacological applications where the external stimulus may be a step function or a peak (bolus administration of a drug, typical for oral administered drugs and intravenous injections) or a continuous stimulus (pharmacological administration of an infusion). More complex tasks may be calculated, for instance, the expression pattern of a network is supposed to be different between day and night time (e.g. according to the circadian rhythm). Also such a desired dynamic network state change can be achieved with external stimuli.

## Results

To illustrate the broadness of our approach we examine two different areas of pharmacological interventions in detail: The results first focus on platelets: Here the fragile balance between platelet activation and hence blood clotting and platelet inhibition with resulting normal blood flow is critical for health and an imbalance in platelet regulation is central to pathological conditions such as stroke and heart failure, still the major causes of death. Hence, manipulation and fine-tuning of external stimuli, in particular anticoagulants, is of high pharmacological interest for medicine and our method is helpful and applied here, giving an important example for pharmacological intervention. The next subection shows that also fine-tuned differentiation processes such as T-cell differentiation can be treated with our method so that differentiation strategies for cell culture experiments or gene knockout and siRNA interference experiments can be modeled and their effects calculated as well as predicted.

### Analysis of the aggregation of platelets

For pharmacological illustration, what to gain by Jimena2 in a concrete application, we look at the aggregation of platelets. We provide here also experimental data validating our work. For this purpose, we use the so called SQUAD model that can be found in the supplementary information of [9]. The model is fitted to experimental data with the software Potterswheel that is mentioned in the Discussion. A schematic of the network can be seen in Fig 1. For our Matlab implementation, we have the following numbers of the nodes. We have P2Y12 is node 1, P2Y1 is node 2, Ca is node 3, Rap1 is node 4, Akt is node 5, Int is node 6, Src is node 7, PI3K is node 8, PTP is node 9, Throm is node 10 and ThromR is node 11. According to [9] a high integrin (Int) activity is associated with irreversible platelet aggregation. We have identified two steady states. First one is (0 0 0 0 0 0.91 0 0 0 0.1 0) which has a high integrin activity and thus it is associated with irreversible platelet aggregation. The second one is (0 0 0 0 0 0 0 0 0 0.1 0) which has a low integrin activity and thus it is associated with reversible platelet aggregation. Note that the values of the steady state close to zero are rounded to zero. In [9], we find that adenosine diphosphate (ADP) activates the irreversible aggregation by stimulating the G-protein-coupled receptors (P2Y1 and P2Y12). For all our calculations we use a time horizon *T* = 100 time units and a discretization *dt* = 0.01 to obtain a stable numerical solution of the system of ordinary differential equations that has the correct asymptotic behavior.

In the following experiment, we would like to induce a switch from the reversible aggregation to the steady state which is associated with irreversible aggregation to demonstrate that the framework is able to find a biological meaningful solution. We have the same system of equation as in [9, SQUAD model]. However the activation of P2Y1 and P2Y12 is modeled as follows. We have

$$\begin{array}{c} \begin{array}{cc} \frac{d}{dt}x_{1} & =-1.3x_{1}+0.6u_{1} \end{array} \end{array}$$

$$\begin{array}{c} \begin{array}{cc} \frac{d}{dt}x_{2} & =-0.47x_{2}+0.6u_{2} \end{array} \end{array}$$

where *u*_1_ and *u*_2_ are an activation stimulus like ADP for example for P2Y12 and P2Y1, respectively. As by the fitting of the data, the nodes’ activity levels are not restricted to the interval [0, 1] and thus we do not have to multiply the activity stimulus by an additional term considering the maximum activity level of the node forcing the activating stimulus term to zero if the node’s activity level reaches its maximum. Furthermore, we equip PTP with an activating stimulus, ThromR with an inhibiting stimulus and Akt with an inhibiting stimulus. We use the output from Algorithm 3 for maxNum = 2, tol = 0.1, *η* = 0.5 and *τ* = 0.9 as an input for Algorithm 2 for *α* = 0.1 that converges here faster than the projected gradient method. From Algorithm 3, we obtain that applying stimulus *u*_1_ for 22 time units induces already a switch. From Algorithm 1, we also obtain that additionally external stimulus *u*_2_ steers the system towards irreversible platelet aggregation. This is in accordance with previous results [9] on the activation of the two G-protein coupled receptors, i.e. that they together trigger irreversible platelet aggregation.

**Fig 2:**
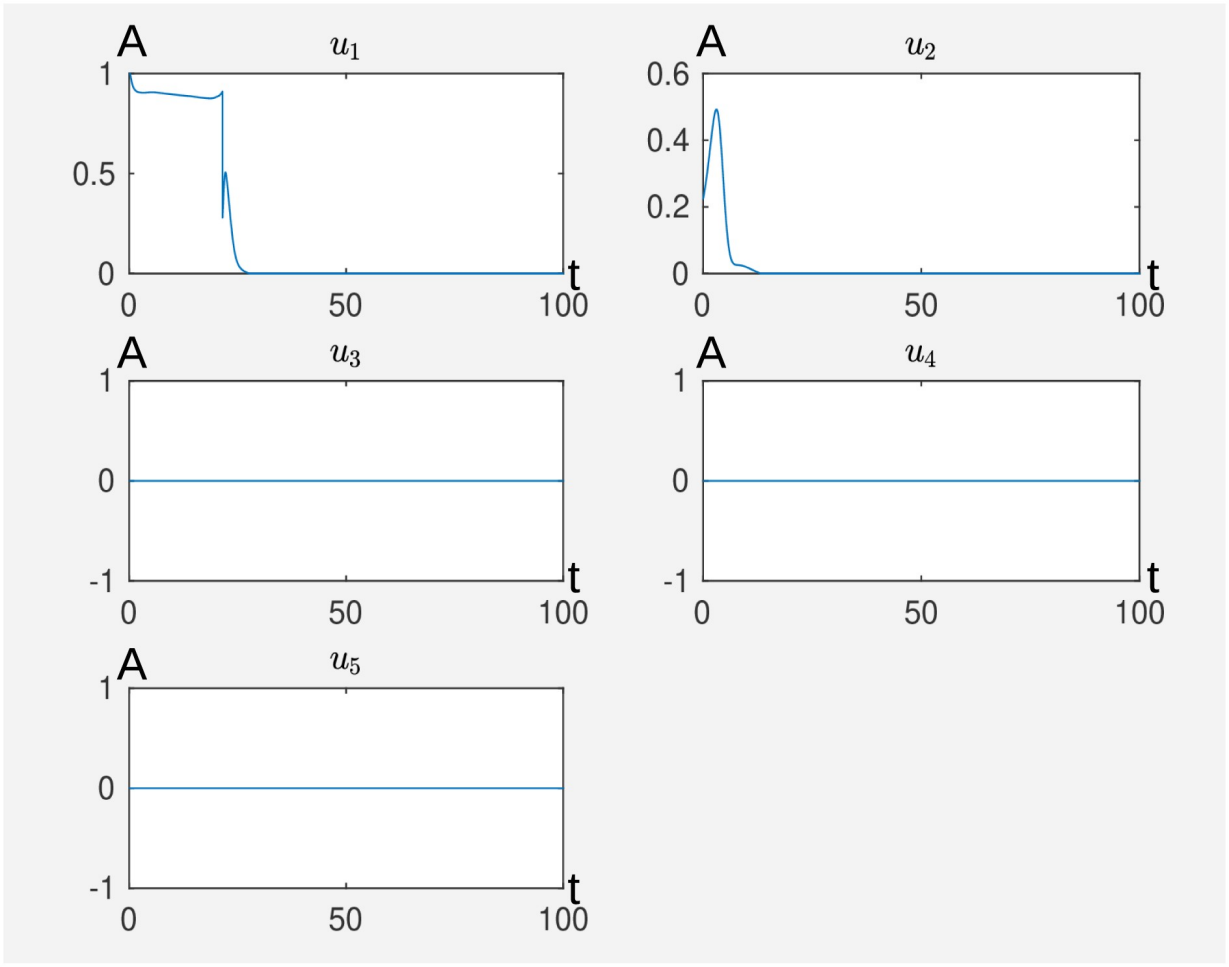
We see the active external stimuli *u*_1_ (activating P2Y12) and *u*_2_ (activating P2Y1) where the others are not active. The others are *u*_3_ activating PTP, *u*_4_ inhibiting ThromR and *u*_5_ inhibiting Akt if active. The time is on the abscissa (t) and the activity level (A) is on the ordinate. The original Matlab output is without axis labeling.

**Fig 3:**
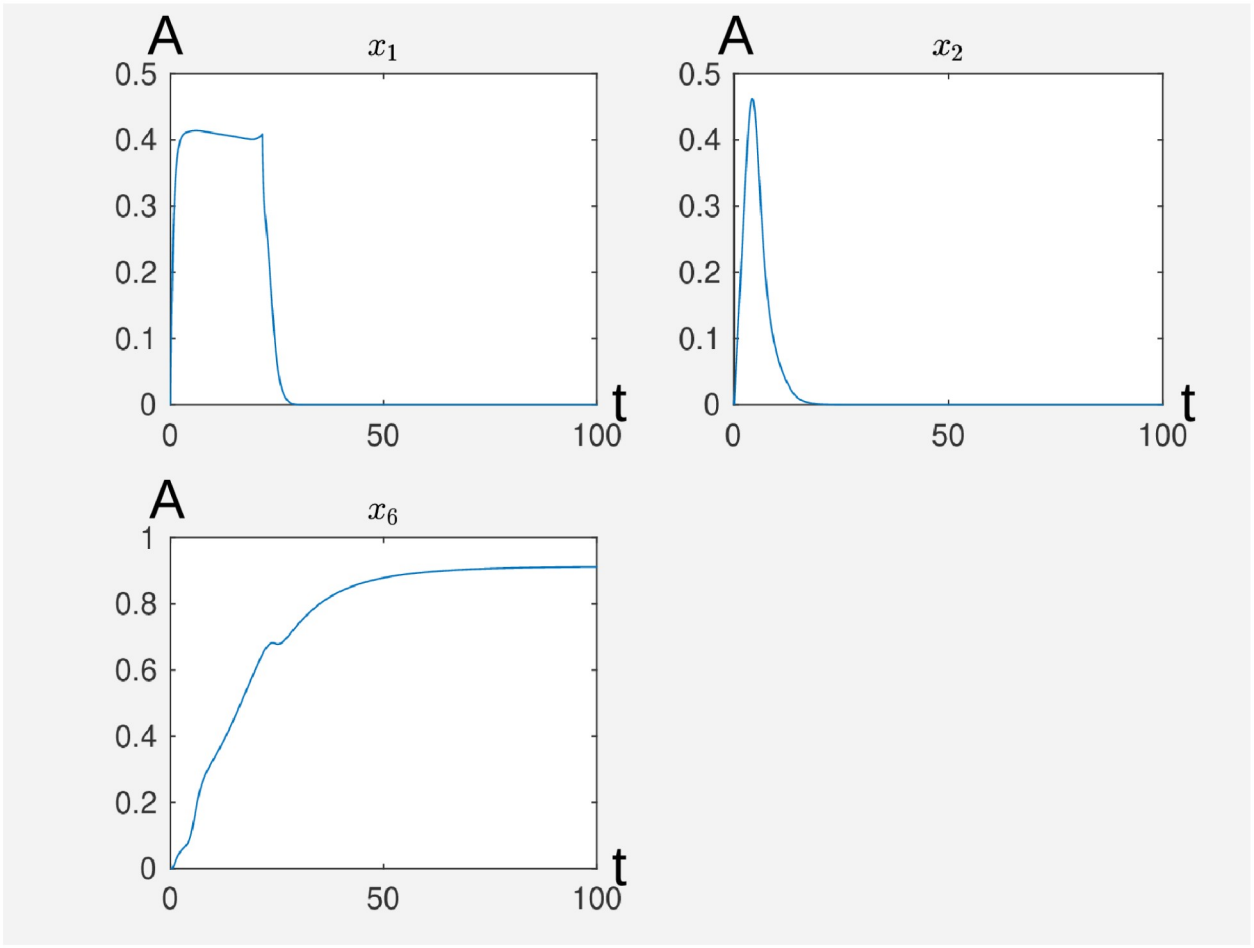
We have the activity levels of P2Y12 (*x*_1_), of P2Y1 (*x*_2_) and of integrin (*x*_6_) where we see the decay of the receptors’ activity while the activity level of integrinconverges to 90%. The time is on the abscissa (t) and the activity level (A) is on the ordinate. The original Matlab output is without axis labeling.

Numerous publications detail results of different pharmacological modulations of platelets and their activation networks [16, 17] and the effects of individual receptor stimulations in our model correlate well with these data. However, our method allows now to exactly calculate external manipulation of the two receptors with or without any other pharmacological intervention point of choice, for instance also inhibition of the cascade, such that either the aggregation threshold is exactly reached, never reached or goes well beyond it. To know and calculate accurately these different pharmacological strengths are critical for fine-tuning antithrombotic therapy and are provided here for the first time in a calculation package. In practice, various antithrombotic drugs exist which often stimulate, besides their cognate receptor, also other targets. Again, our method for the exact calculation of the network effects of combination treatment provides thus here a rational basis for optimal therapeutic intervention. The results from Algorithm 2 can be seen in Figs 2 and 3. Pharmacologically most often a bolus or continuous infusion is given (orally or intravenous administered drug). This bolus function or continuous drug level can also easily be considered as external stimulus or even a combination therapy involving both:

Notice that once the activity levels of the external stimuli are related to a concentration or intensity (model fitting of the coupling constants *σ*_*kj*_, *η*_*kj*_ of the external stimuli with the nodes) in the real experiment and the most effective external stimuli are identified (external stimuli with a non-zero time curve), the exact time curves of the external stimuli are not that important with respect to inducing a switch, see the experiment corresponding to Figs F and G in S1 File and the corresponding text below Fig G in S1 File. We can concentrate on the most effective external stimuli and can try different time curves, like constant ones, that are easier to implement in a real experiment and still perform the desired switch.

### A switch between two different types of T-helper cells

**Fig 4:**
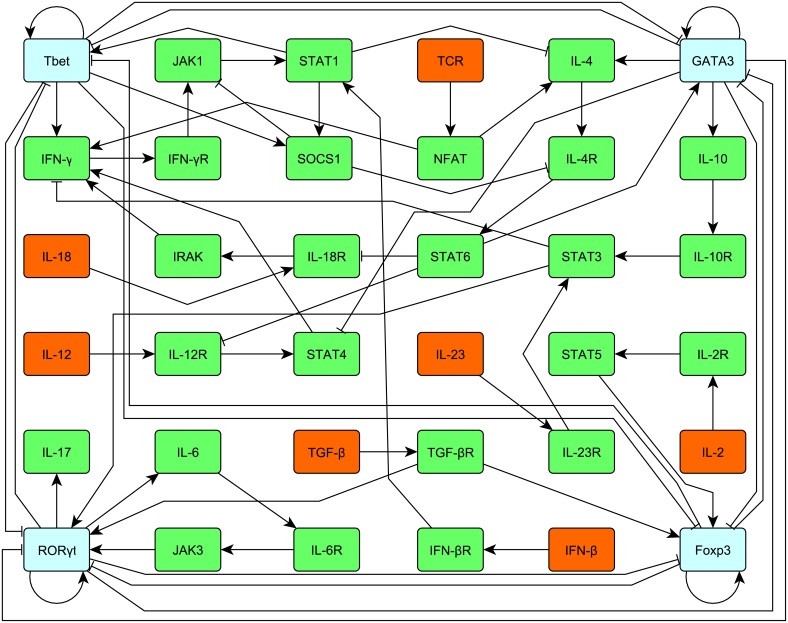
Schematic of the CD4+ T-helper and Treg network from [10, Figure 2].

There are other manipulations of the network that also cover an important application area, in particular regarding differentiation, cell maturation (e.g. in immunity), effects of gene knockdowns etc. To study this in a suitable example we chose T-cell maturation. In this subsection, we analyze a network modeling the differentiation of different types of T-helper cells as well as CD4+ Foxp3+ regulatory T cells (Treg). The model whose schematic is given in Fig 4 is investigated in [10] with respect to its steady states and it is shown there that this network has five steady states. There is one steady state for each special type of CD4+ T-helper cell, namely Th0, Th1, Th2, Th17 and, in addition, Treg.

We use (2) where all the parameters are set to 1 except *h* = 50, see [10, Table 1]. We introduce the following external stimuli. External stimulus *u*_1_ activates IFN-*β*, external stimulus *u*_2_ activates IL-12, external stimulus *u*_3_ activates IL-18, external stimulus *u*_4_ activates IL-2, external stimulus *u*_5_ activates IL-23, external stimulus *u*_6_ activates the TCR, external stimulus *u*_7_ activates TGF-*β* and external stimulus *u*_8_ inhibits ROR*γ*t, *u*_9_ inhibits IL-6 and *u*_10_ inhibits IL-6R which is the receptor for IL-6. With this set of external stimuli, we intend to induce a switch from Th17 with the corresponding steady state of the network [0, 0, 0, 0, 0, 0, 0, 0, 0, 0, 1, 0, 0, 0, 0, 0, 0, 0, 0, 1, 1, 0, 0, 1, 0, 1, 0, 0, 0, 0, 0, 0, 0, 0, 0, 0] which serves as initial state for the network to Treg with the corresponding steady state [0.9998, 0, 0, 0, 0, 0, 0, 0, 0, 0, 0, 0, 0, 0, 0, 0, 0, 0, 0, 0, 0, 0, 0, 0, 0, 0, 0, 0, 0, 0, 0, 0, 0, 0, 0, 0], see Table 2 in [10] for all the steady states. We remark that the model is optimized to get either a number close to 1 if the corresponding node is active or 0 if the corresponding node is inactive in order to obtain a clear expression pattern for each type of T-cell.

**Fig 5:**
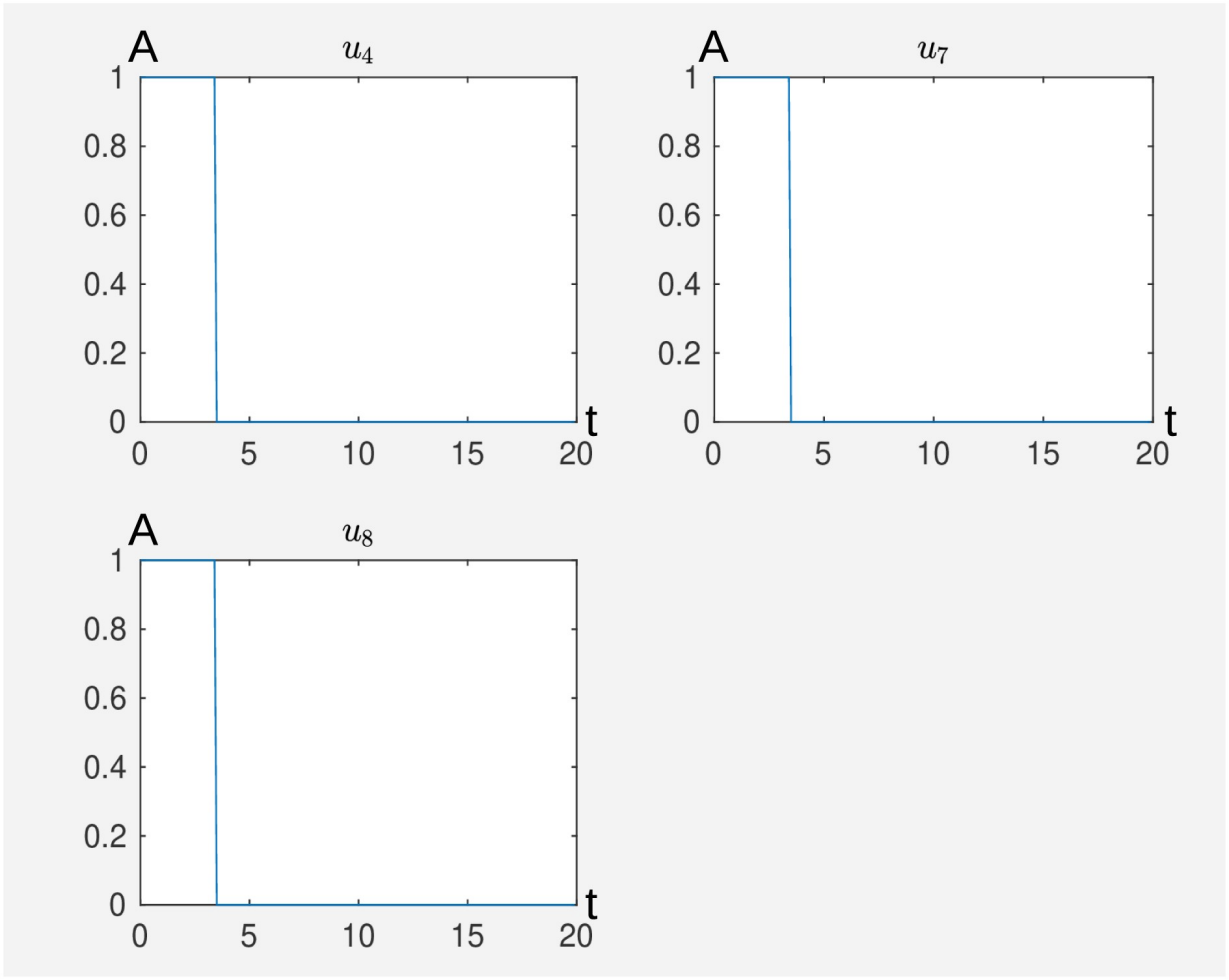
External stimuli that cause a switch from Th17 to Treg where in this figure there is a minimal set of external stimuli that performs the desired switch calculated by Algorithm 3 where *u*_4_ activates IL-2, *u*_7_ activates TGF-*β* and*u*_8_ inhibts ROR*γ*t. The time is on the abscissa (t) and the activity level (A) on the ordinate. The original Matlab output is without axis labeling.

**Fig 6:**
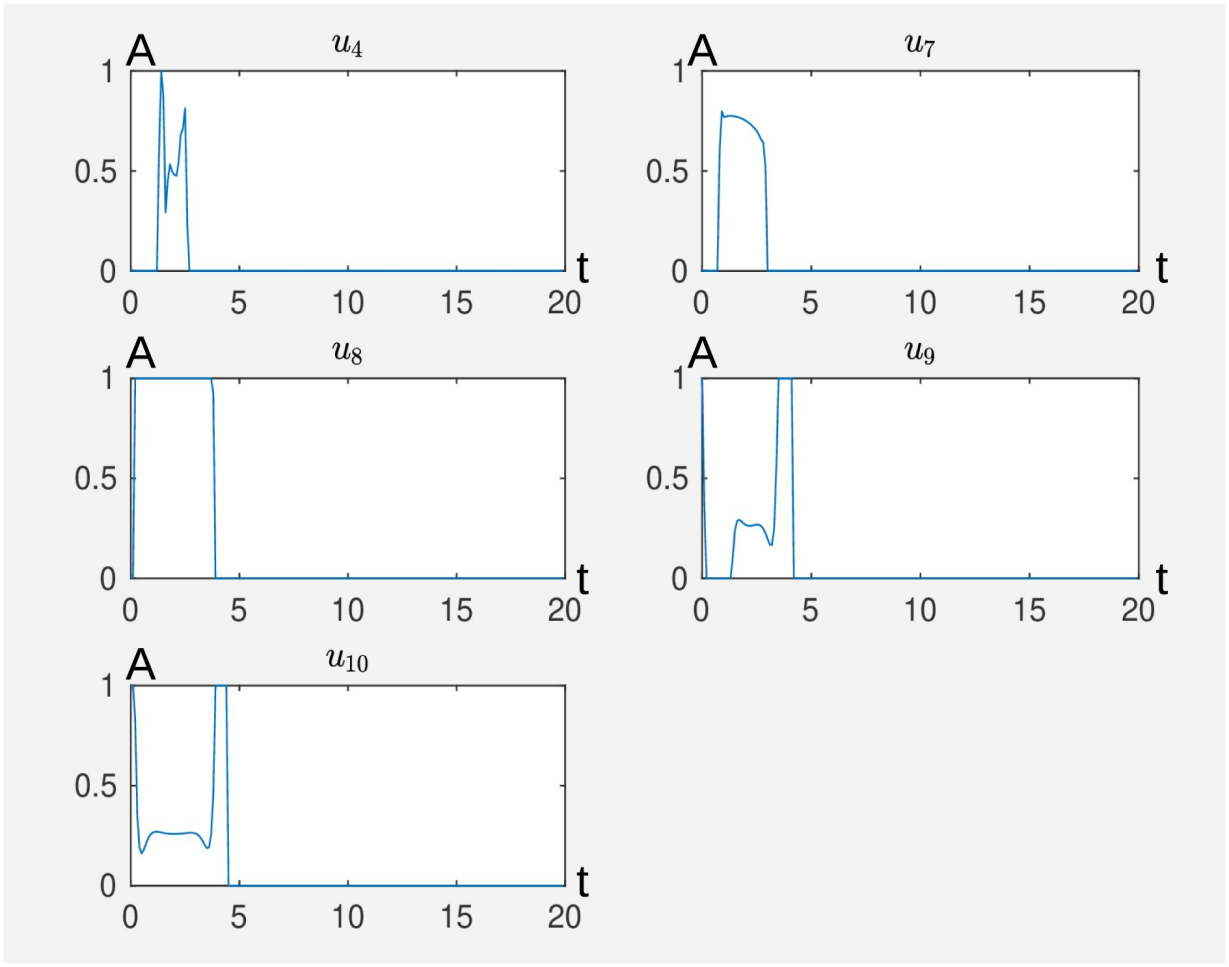
External stimuli that cause a switch from Th17 to Treg where in this figure we calculated with Algorithm 1 further external stimuli that support the desired switch caused by the external stimuli depicted in Fig 5 where *u*_4_ activates IL-2, *u*_7_ activates TGF-*β*, *u*_8_ inhibits ROR*γ*t, *u*_9_ inhibits IL-6 and *u*_10_ inhibits IL-6R. The time is on the abscissa (t) and the activity level (A) on the ordinate. The original Matlab output is without axis labeling.

In Fig 5, we see the result from Algorithm 3 for maxNum = 4, tol = 0.1, *η* = 0.5 and *τ* = 0.9. We see that according to our model, inactivation of ROR*γ*t and activation of IL-2 and TGF-*β* for about 4.5 time units performs the desired switch. We take the result from Algorithm 3 as initial guess ^0^*u* for Algorithm 1 for the parameters *α* = 0.5, *T* = 20 and *dt* = 0.1. In Fig 6 we see the results, i.e. that the inactivation of IL-6, *u*_9_ and IL-6R, *u*_10_ supports the switch, that means that the network switches faster to the Treg cell. Furthermore, we can deduce more structure from the model that first the inhibiting stimuli, *u*_8_, *u*_9_, *u*_10_ are active before the activating external stimuli *u*_4_ and *u*_7_ come into play. This can be interpreted in the way that activation of the T-cell by IL-2 and TGF-*β* is only efficient to induce the desired switch from Th17 to Treg if the specific Th17 expression pattern is knocked down to a certain degree. For this purpose it is sufficient to knock down ROR*γ*t that can be supported by the knock down of IL-6 and IL-6R. We stress that by virtue of the self activation of ROR*γ*t, a direct knock down of the node ROR*γ*t is necessary to induce the switch according to our model.

A switch from Th17 to Treg has, indeed, been described e.g. in the tumor setting [18]. Here, soluble factors contained in ovarian cancer ascites were capable of mediating the transdifferentiation from Th17 to Treg. While the Foxp3-inducing effect of the cancer ascites was mimicked by the addition of recombinant TGF-*β*, the ascites and TGF-*β* differed in that TGF-*β* alone induced IL-17A together with Foxp3 expression. Therefore, the cancer ascites must contain additional factors which suppress ROR*γ*t expression—as we show here a prerequisite to complete the switch from Th17 to Treg.

Furthermore, our predictions, for example those of Figs 5 or 6, regarding which external stimuli induce a switch can be used for a data bank search as follows: The soluble factors of the ascites can be analyzed and the information can be stored in a data bank. Then, by our calculations, we have generated candidates for which we can look out for in our data bank to explain the observed switch: More specific, we can search for proteins that have a DNA-binding site for an IL-2 enhancer or the IL-2 promotor. In addition, we can look for proteins that have IL-2-like domains. On the other hand, we can search in the data bank for proteins that bind to a silencer of ROR*γ*t or have interacting domains that are able to bind to sites of ROR*γ*t and thus biologically inactivate ROR*γ*t.

In addition, we remark that the exact time curve in Fig 6 is not important for the switch, compare with Fig 5. Hence also typical drug action curves (step function or exponential decay) work also here. For details see the corresponding remark about this issue in S1 File, which is the corresponding experiment to Figs F and G in S1 File and the remark before Fig 2 in the present manuscript.

In our experiments which we perform with the network proposed in [10], we notice that once the network has taken a steady state corresponding to a T-cell type Th1, Th2, Th17, Treg, the corresponding attractor is quite robust under perturbations of our external stimuli and is stable with respect to switches between different cell types. However, according to our model presented here in the external stimuli framework for desired switches, roughly spoken, mostly one has to knock down the lineage-specifying transcription factor of the cell type in which one starts and activate the related cytokines and lymphokines of the desired cell type in order to induce the desired switch of cell types into any desired one, i.e. Th1, Th2, Th17, Treg. The switch from Th17 to Treg differentiation is explained in detail and specific stimuli (effects of IL-6, IL-2, receptor stimulation e.g. IL-6R) correlate well with the available experimental data. However, in experiments the best combination of external stimuli to switch the immune cell phenotype is far less clear and generally tested empirically in numerous experiments as the network effects are not intuitively clear. Here our method provides unprecedented clarity and saves time and experiments. Moreover, a further gain from our in silico model is that also the transdifferentiation to the other cell types (e.g. Th1 to Th2) are perfectly charted by our approach.

## Discussion

We first discuss differences to other software existing to analyze regulatory networks and illustrate how to combine our provided software package with other tools. In particular, there is no calculation of optimal modulation of networks by external stimuli available. However, the selection of an optimal stimulus for a specified number of nodes to be modulated into a new state is central for pharmacological interventions. Moreover, planning of knockdowns, RNAi experiments for instance, or any kind of other inhibition, excitation or modulation of the network would highly profit from such an application and this is offered here. None of the tools currently available does this.

On the other hand each of them allows an efficient description of the system state including suitable differential equations using either accurate or heuristic solutions. Iterative model refinement can be done including studying the effect of different receptor inputs, activations and inhibitions. However, none of these tools allows to directly calculate the optimal manipulation of the network for changing to a prespecified new system state. Instead there would be numerous trials and iterative steps necessary using any of the software below to achieve this.

We start with **SQUAD** (https://sbos.eu/docu/docu/SQUAD/doku.php.htm). One can utilize SQUAD to analyze steady states of a network once one has the topology of the network that means the interaction graph. Additionally, one can perturb the network for example by activating receptors or by changing decay or gain of a node’s activity. However, this software does not provide a systematical procedure how to search for perturbations to steer the network to a desired state. Therefore our concept of providing a framework to calculate perturbations by techniques of mathematical optimization to induce a switch between an initial steady state and a desired final steady state supplements the pioneering idea of SQUAD implemented in a software.

**JIMENA** (https://www.biozentrum.uni-wuerzburg.de/bioinfo/computing/jimena-c/) is a software package which focuses on an efficient numerical steady state analysis. Therefore, this package as well as SQUAD serves as an excellent tool to figure out the steady states of a network between which one would like to switch. Once the steady states are found, our framework proposed in the present work can be utilized for the calculation of the desired switch.

**CELL NET ANALYZER** (https://www2.mpi-magdeburg.mpg.de/projects/cna/cna.html) provides, besides metabolic network analysis and steady state analysis, a method to calculate minimal intervention sets that provide a network with desired steady states. However, this means that one gets a list of knockins and knockouts of nodes of the network such that the network attains the desired steady states. This is a very important tool, especially in the case of lacking experimental information or conflicting information about the interaction of the considered agents, to design the topology of the network in order to obtain a network that provides the steady states sought. Thus this method is intended to change the topology of the network that means the interaction graph while our approach deals with a fixed topology of the network during all calculations. CELL NET ANALYZER is hence well suited for metabolic modeling, modeling of signalling networks and the upstream use of SQUAD or JIMENA to design an appropriate network but not for precalculation of the best and most efficient stimulus combination to achieve a desired switch between two different system states.

**ODEFY**(https://www.helmholtz-muenchen.de/icb/software/odefy/index.html) is a tool in order to convert boolean networks into networks whose dynamic is described by ordinary differential equations (ODEs) which are the basic models in our framework. Therefore ODEFY is a key tool to transform network topologies into an ODE model which is essential for our framework, especially in the case of networks with many nodes and couplings between them. Thus ODEFY allows the swift formulation of the differential equation system, however, there is no systematic approach to identify the correct combination of external stimuli.

Other software applications (two examples given) allow to define differential equations for modeling biological systems flexibly. In principle pharmacological modeling is cumbersome in practice. For this purpose, we provide in addition a straight forward tool to 1) define and 2) apply functions $u_{j}:R_{0}^{+}\to [0,1]$, *j* ∈ {1, …, *m*}, m∈N as pharmacological parameters.

Specifically, **COPASI** toolbox (http://copasi.org/) focuses on the modeling and on simulating of biochemical reaction networks, especially simulation of time curves and steady states, parameter fitting of an underling model to experimental data, sensitivity analysis and dynamical behavior characterization. Besides steady state analysis, a further step of network analysis is to compare the simulated curves with experimental data. Therefore COPASI is a very important tool to analyze different models that fit the data best after fitting of the model’s parameters. Thus COPASI can have a big contribution to pharmacological modeling by first fitting parameters and then choose the model that fits the data best which is essential for dose rate calculations, for example. As our framework does not depend on special network equations that are set up from the interaction graph, our presented framework extends the great range of functions in a useful way. Moreover, COPASI is maybe even more powerful then ODEFY and wide ranges of parameters can be tested swiftly. Nevertheless, to find a proper combination of external stimuli to change the system state is a challenge, cumbersome and not systematic.

The software tool **POTTERSWHEEL** (http://www.potterswheel.de/Pages/index.php) can be used for modeling with ODEs and best parameter fit to experimental data. For this purpose, one can additionally include external driving functions or vary parameters. This can be used to adapt parameters of the system of ODEs with which one would like to model a particular real network such that the theoretical data fits best to the experimental data. Moreover, POTTERSWHEEL is very efficient in model testing and model refinement. However, for our task, pharmacological network modulation of the change of network state by the best, most efficient combination of external stimuli, the tool allows to quickly verify that a given combination is right, but to find it is difficult and needs numerous iterations. On the other hand, individual parameters are fast characterized regarding their sensitivity and importance for network behavior. Therefore, as COPASI, the software POTTERSWHEEL is also very helpful to figure out an appropriate model and can be extended by our framework with a further useful feature.

Next, we explain why there is a specific need for the software, mathematically speaking. The last two software packages are well known examples and perform, among other things, a fit of model parameters of a system of coupled ODEs. This is similar to our framework. However, the main difference between a parameter fit and our framework is, that the functions *u*_*j*_, *j* ∈ {1, …, *m*} are time dependent while a parameter is constant. Therefore we extend the idea of parameter fit, which is a special application of mathematical optimal control theory, to a more general framework that also includes time dependent parameter fitting. In our case, the time dependence of the functions *u*_*j*_ is important as they are switched on for a certain time and after the switch off, the network can relax to the desired state such that we can verify, after the application of certain external stimuli, that each node is close to its desired state which would not be possible with a constant parameter as the network could not relax with constitutively activated external stimuli.

Our framework provides the most important external stimuli that are responsible for a desired switch. By this procedure, we can reduce the number of pharmacological intervention points to a small but very effective number which is in favor of pharmacological treatments. This issue is also not covered by classical parameter fitting that is provided by COPASI and POTTERSWHEEL. However, as we mentioned before, our framework of systematically finding external stimuli that steer the network from an initial steady state to a desired one is not restricted to a special type of ODE model. Therefore the provided Matlab files can also be integrated into existing software packages like COPASI or POTTERSWHEEL to extend their already great and helpful tools for model analysis with respect to experimental data.

In the following we discuss some general properties of the proposed method. We first discuss the sensitivity of the proposed framework to model parameters that have to be concerned for modeling [19]. We remark that the steady states depend more on the interaction graph, that means how the nodes are connected with each other and less on the model parameters. However the values of each component of the steady state depend on the model parameters. That means as long as one is only interested in the qualitative information which nodes have to be affected by external stimuli for a desired switch and not exact durations of external stimuli, the proposed method will give the same result for a wide range of values for the model parameters. On the other hand, if we have a model that fits the data very well, the proposed framework gives the possibility for a very careful network analysis as exact thresholds for certain protein concentrations can be calculated and quantitative predictions can be made. However this is very intuitive as exact predictions require a detailed understanding of the biological system which means that a detailed model is required which fits to the experimental data sufficiently well. In other words the better the model describes the considered experiment, the more reliable are the results from the proposed method. This means the key point for reliable results is still the model. The proposed framework just reveals the information needed to induce a desired network behavior and that is encoded in the model.

The capability of the framework to reveal information in the model about external stimuli and their influence on the network is also a tool for validating a model. For example, if a certain set of external stimuli induces a switch in a real experiment, but this is not obtained by calculations with a corresponding model, it can be a hint that the model structure or its parameters are not set or fitted such that the model is sufficiently exact for the considered issue. Then one has a further option for testing the model by varying model parameters until the calculations give the same external stimuli that are found in the real experiment. If this is still not achievable, the model topology and components have to be reexamined and a new simulation cycle has to be started with a carefully revised model.

We remark that the proposed framework systematically includes the external stimuli into the model. This is a very important brick for exact modeling by fitting software for the following reason. The data measured in experiments is usually the adaption of the observed system to external stimuli. That means the data is the variation of observed quantities upon external stimuli. These external stimuli can be set as time dependent functions and thus the information on the action of the external stimuli can be systematically and exactly included into the fitting process. By this way several data sets can be created in experiments such that a fitting software can calculate the model parameters such that the theoretical time curves fit best to the experimental time curves of the corresponding molecular agents. By measuring the distance between the theoretical time curves and the experimental ones, one can compare different models that all have been fitted to the same experimental data and take this one with the smallest distance. This model is the best fitting one and thus describes the considered experiment best which means contains the most reliable information about the considered biological system. Keep always in mind that there is more than just one possibility to fit the model to data. We illustrate one possibility where our proposed framework fits well in. In particular, model selection is not only based on the distance between data and model prediction, but also on the number of free parameters used to fit the data as quantified by information theoretical measures such as AIC (Akaike Information Criterion) or BIC (Bayesian Information Criterion).

Another important issue that needs to be discussed is the choice of the parameter *α* in (6). By increasing *α* one increases the costs for the action of an external stimulus. This means by solving the optimal control problem (8) several times with increasing *α* filters out external stimuli whose input have the biggest influence on the desired switch. A systematical strategy for the choice of *α* in order to figure out only a small number and thus most effective external stimuli can be the following. We can increase *α* until the result consists of only non-active external stimuli. Then we can set *α* between the biggest value where there have been non-zero external stimuli and the smallest *α* where there are only non-active external stimuli in the solution of the corresponding optimal control problem. This can be repeated until the interval between these two numbers is below a given tolerance.

We remark that perturbations by our external stimuli framework proposed in (2) or (3) differ from just perturbing the initial values of the network. In the proposed framework of external stimuli, the duration of application can be varied that cannot be done in the case of perturbing initial values. Therefore activating or inhibiting external stimuli can act sufficiently long such that inertial network effects can be overcome, for example activated nodes can decay and inactivated nodes can be activated sufficiently long by others until they reach a certain threshold such that the network relaxes into its desired state. For example, if one takes the steady state associated with the Th17 cell type as an initial state of the network where additionally the initial value for ROR*γ*t is set to zero and the initial value for IL-2 and TGF-*β* is set to one according to the external stimuli proposed in Fig 5, then the network relaxes back to the steady state corresponding to the Th17 cell type. This results since just the perturbation of the initial value does not reach a necessary threshold of the activation levels which would cause the relaxation of the network to the desired state. Thus these perturbations are not sufficient to perform the desired switch. This shows that the inertial network effects can overcome perturbations of the initial values but not if the perturbations of the corresponding nodes last sufficiently long and go beyond a perturbation of an initial state, then a desired switch can happen. Furthermore, in contrast to our external stimuli framework, it is difficult to implement perturbations of the initial state of a real biological network in an experimental setting because this means, based on the example of the Th17 cell mentioned above, that one has to set up a cell with an expression level where the expression of ROR*γ*t is low while at the same time the expression level of IL-17 and IL-6 is high. This might not be possible as the transcription of IL-17 and IL-6 directly depends on the presence of ROR*γ*t, see Fig 4.

Table 1 summarizes the discussion including validating evidence from previous publications. Similarly, our provided model can be used to calculate external stimuli for other switches between any two of Th0, Th1, Th2, Th17 or Treg and systematically be compared to experimental work investigating such switches (in particular different T-cell maturation processes [20–22]).

**Table 1:** Validation for the T-helper cell model.

Experimental evidence	Relation to the calculation of T-helper cell subsection
ROR*γ*t antagonists (e.g. digoxin, GSK805) inhibit Th 17 cell maturation [23, 24] (with GEO data sets)	Inhibition of ROR*γ*t necessary for the desired switch from Th17 to Treg
TGF-*β* is Foxp3-inducing, but not switch inducing from Th17 to Treg [18]	Besideds TGF-*β*, additional factor required for the switch from Th17 to Treg, like ROR*γ*t inhibitors or IL-2/IL-2 activators

Interventions and experimental evidence supporting topology and dynamic model of T cell maturation.

For the experimental realization of our predictions, cytokines and cytokine receptors can be readily inhibited e.g. by monoclonal antibodies or the receptors stimulated by recombinant cytokines. For the T-cell lineage-specifying transcription factors like ROR*γ*t therapeutic targeting is per se more difficult. However, in case of ROR*γ*t with digoxin as an ‘old’ drug and with newly developed compounds like GSK805 specific inhibitors are available also for clinical use [23, 24]. In addition a knock out of lineage-specifying transcription factors is also possible, but the knock out changes the topology of a network and thus is not covered by our model so far. Nevertheless knocking out the T-cell lineage-specifying transcription factor and activating cytokines for cell polarization into the desired T-cell type might also work to induce the desired switch. This intuition comes from the fact that each lineage-specifying transcription factor is connected to each other, see Fig 4, which might provide a robustness with respect to a knock out of a lineage-specifying transcription factor. Our tool charts novel routes and intervention combinations while for already well-charted intervention points our predictions have been confirmed by independent approaches, in particular experiments. This is the case both for platelet modeling (Subsection “Analysis of the aggregation of platelets”) as well as T-cell differentiation (Subsection “A switch between two different types of T-helper cells”). The challenges in both systems become also clear: For the generally well-charted platelet receptor network the exact calculation of combination effects by the tool is new and with potential clinical implications in particular for anticoagulation therapy and modulating platelet aggregation, thrombosis and hemostasis. In the T-cell network the tool confirms not only well-known interleukin and receptor effects but charts and suggests new, not so well explored, routes for achieving switches in T-cell differentiation. Of course, the tool can also be applied to any other signaling network to study its external and/or its pharmacological modulation -provided its topology is sufficiently well known.

### Conclusion

A detailed mathematical extension of a regulatory network to controlled regulatory networks with external stimuli was presented. Different methods for a systematic calculation of the external stimuli inducing the desired switch were illustrated. A software tool was developed. The considerations presented in the presented work hold for any model consisting of well-posed ordinary differential equations, like chemical reaction networks [13–15]. Since the model equations are an input of the software tool, the provided implementations can be applied to a wider class of models than the ones used in the present work. The application of the proposed framework how to analyze a controlled regulatory network with respect to finding an optimal selection of external stimuli which cause a desired switch between two different steady states of a regulatory network was demonstrated with examples. Biological validation was made by considering a regulatory network modeling platelet aggregation and identifying and fine-tuning the receptors that were associated with triggering the irreversible aggregation. Furthermore, a switch from a T-helper cell type Th 17 to Treg is predicted by our theoretical investigations and discussed to validate this statement.

Furthermore, the proposed algorithm strategy directly calculates the best intervention points and strength of intervention. We show how it extends already available software such as Potterswheel, ODEFY or COPASI by an optimal targeting method allowing to calculate best combinations of external interactions. It has many applications for improved pharmacological treatment, recognition of the optimal target or drug combination as well as for molecular interventions.

## Supporting information

S1 File Mathematical and algorithmic details for solving optimal control problems, small example demonstrating the scope and the use of the presented optimal control framework for calculating appropriate intervention points. (PDF)

## References

[1] Mendoza Luis and Xenarios Ioannis. A method for the generation of standardized qualitative dynamical systems of regulatory networks. *Theoretical Biology and Medical Modelling*, 3(1):13, 2006 10.1186/1742-4682-3-13 16542429 PMC1440308

[2] Cara Alessandro Di, Garg Abhishek, Micheli Giovanni De, Xenarios Ioannis, and Mendoza Luis. Dynamic simulation of regulatory networks using SQUAD. *BMC bioinformatics*, 8(1):462, 2007 10.1186/1471-2105-8-462 18039375 PMC2238325

[3] Karl Stefan and Dandekar Thomas. Jimena: efficient computing and system state identification for genetic regulatory networks. *BMC bioinformatics*, 14(1):306, 2013 10.1186/1471-2105-14-306 24118878 PMC3853020

[4] Kratchmarova Irina, Blagoev Blagoy, Mandana Haack-Sorensen, Kassem Moustapha, and Mann Matthias. Mechanism of divergent growth factor effects in mesenchymal stem cell differentiation. *Science*, 308(5727):1472–1477, 2005 10.1126/science.1107627 15933201

[5] Schuldiner Maya, Yanuka Ofra, Joseph Itskovitz-Eldor, Melton Douglas A., and Nissim Benvenisty. Effects of eight growth factors on the differentiation of cells derived from human embryonic stem cells. *Proceedings of the National Academy of Sciences*, 97(21):11307–11312, 2000 10.1073/pnas.97.21.11307 PMC17196 11027332

[6] Yamanaka Shinya and Blau Helen M. Nuclear reprogramming to a pluripotent state by three approaches. *Nature*, 465(7299):704–712, 2010 10.1038/nature09229 20535199 PMC2901154

[7] Martin Shaun, Lamb H.K., Brady C., Lefkove B., Bonner M.Y., Thompson P., Lovat P.E., Arbiser J.L., Hawkins A.R., and Redfern C.P. F. Inducing apoptosis of cancer cells using small-molecule plant compounds that bind to GRP78. *British journal of cancer*, 109(2):433–443, 2013 10.1038/bjc.2013.325 23807168 PMC3721410

[8] Sreevalsan Sandeep, Joseph Sonia, Jutooru Indira, Chadalapaka Gayathri, and Safe Stephen H. Induction of apoptosis by cannabinoids in prostate and colon cancer cells is phosphatase dependent. *Anticancer research*, 31(11):3799–3807, 2011 22110202 PMC3280884

[9] Mischnik Marcel, Gambaryan Stepan, Subramanian Hariharan, Geiger Jörg, Schütz Claudia, Timmer Jens, and Dandekar Thomas. A comparative analysis of the bistability switch for platelet aggregation by logic ODE based dynamical modeling. *Molecular BioSystems*, 10(8):2082–2089, 2014 10.1039/c4mb00170b 24852796

[10] Mendoza Luis and Pardo Fátima. A robust model to describe the differentiation of T-helper cells. *Theory in biosciences*, 129(4):283–293, 2010 10.1007/s12064-010-0112-x 20922578

[11] Lakin Nicholas D. and Jackson Stephen P. Regulation of p53 in response to DNA damage. *Oncogene*, 18(53), 1999. 10.1038/sj.onc.1203015 10618704

[12] Weber Gregor G., Kortmann Jens, Narberhaus Franz, and Klose Karl E. RNA thermometer controls temperature-dependent virulence factor expression in vibrio cholerae. *Proceedings of the National Academy of Sciences*, 111(39):14241–14246, 2014 10.1073/pnas.1411570111 PMC4191814 25228776

[13] Feinberg Martin. *Foundations of Chemical Reaction Network Theory*. Springer, 2019.

[14] Craciun Gheorghe and Pantea Casian. Identifiability of chemical reaction networks. *Journal of Mathematical Chemistry*, 44(1):244–259, 2008 10.1007/s10910-007-9307-x

[15] Irene Otero-Muras, Yordanov Pencho, and Stelling Joerg. Chemical reaction network theory elucidates sources of multistability in interferon signaling. *PLoS computational biology*, 13(4):e1005454, 2017 10.1371/journal.pcbi.1005454 28369103 PMC5400276

[16] Desborough MJR, Oakland KA, Landoni G, Crivellari Martina, Doree C, Estcourt LJ, and Stanworth SJ. Desmopressin for treatment of platelet dysfunction and reversal of antiplatelet agents: a systematic review and meta-analysis of randomized controlled trials. *Journal of Thrombosis and Haemostasis*, 15(2):263–272, 2017. 27893176 10.1111/jth.13576

[17] Jamasbi Janina, Ayabe Keng, Goto Shinya, Nieswandt Bernhard, Peter Karlheinz, and Siess Wolfgang. Platelet receptors as therapeutic targets: past, present and future. *Thrombosis and haemostasis*, 117(07):1249–1257, 2017 10.1160/TH16-12-0911 28597906

[18] Stephanie Downs-Canner, Berkey Sara, Delgoffe Greg M., Edwards Robert P., Curiel Tyler, Odunsi Kunle, Bartlett David L., and Nataša Obermajer. Suppressive IL-17A+ Foxp3+ and ex-Th17 IL-17A neg Foxp3+ T reg cells are a source of tumour-associated T reg cells. *Nature communications*, 8:14649, 2017 10.1038/ncomms14649 PMC5355894 28290453

[19] Gutenkunst Ryan N., Waterfall Joshua J., Casey Fergal P., Brown Kevin S., Myers Christopher R., and Sethna James P. Universally sloppy parameter sensitivities in systems biology models. *PLoS computational biology*, 3(10):e189, 2007 10.1371/journal.pcbi.0030189 PMC2000971 17922568

[20] Carpenter Robert O., Evbuomwan Moses O., Pittaluga Stefania, Rose Jeremy J., Raffeld Mark, Yang Shicheng, Gress Ronald E., Hakim Frances T., and Kochenderfer James N. B-cell maturation antigen is a promising target for adoptive T-cell therapy of multiple myeloma. *Clinical cancer research*, 2013. 10.1158/1078-0432.CCR-12-2422 PMC3630268 23344265

[21] Hogquist Kristin A., Xing Yan, Hsu Fan-Chi, and Shapiro Virginia Smith. T cell adolescence: maturation events beyond positive selection. *The Journal of Immunology*, 195(4):1351–1357, 2015 10.4049/jimmunol.1501050 26254267 PMC4530466

[22] Hsu Fan-Chi, Belmonte Paul J., Constans Megan M., Chen Meibo W., McWilliams Douglas C., Hiebert Scott W., and Shapiro Virginia Smith. Histone deacetylase 3 is required for T cell maturation. *The Journal of Immunology*, 195(4):1578–1590, 2015. 26163592 10.4049/jimmunol.1500435 PMC4530026

[23] Withers David R., Hepworth Matthew R., Wang Xinxin, Mackley Emma C., Halford Emily E., Dutton Emma E., Marriott Clare L., Verena Brucklacher-Waldert, Veldhoen Marc, Kelsen Judith, et al Transient inhibition of ROR-*γ*t therapeutically limits intestinal inflammation by reducing T H 17 cells and preserving group 3 innate lymphoid cells. *Nature medicine*, 22(3):319, 2016 10.1038/nm.4046 PMC4948756 26878233

[24] Xiao Sheng, Yosef Nir, Yang Jianfei, Wang Yonghui, Zhou Ling, Zhu Chen, Wu Chuan, Baloglu Erkan, Schmidt Darby, Ramesh Radha, et al Small-molecule ROR*γ*t antagonists inhibit T helper 17 cell transcriptional network by divergent mechanisms. *Immunity*, 40(4):477–489, 2014 10.1016/j.immuni.2014.04.004 24745332 PMC4066874

