# Supplementary material for: Analyzing pharmacological intervention points: A method to calculate external stimuli to switch between steady states in regulatory networks
Source: PLoS Comput Biol. 2019 Jul 16;15(7):e1007075. doi: 10.1371/journal.pcbi.1007075 (PMC6660093; doi:10.1371/journal.pcbi.1007075)
Supplement: S1 File — (PDF) [file pcbi.1007075.s001.pdf]

Supplementary material for Analyzing pharmacological intervention points: A method to calculate external stimuli to switch between steady states in regulatory networks

In this supplement we give mathematical and algorithmic details for solving optimal control problems with the purpose of finding effective external stimuli with a desired effect on cells or experiments. Furthermore we demonstrate with a small example the scope and how to use the presented optimal control framework for the calculation of appropriate intervention points. We present here the figures as they are obtained directly from the Matlab scripts so that the reader knows how the output from the scripts will look like. In the results section of the paper, however, we then polished the output further (proper axis labeling according to biological entity and biological interpretation.

1 Solution strategy and algorithms for optimal control problem

In order to solve

$$\begin{aligned} &\min_{x,u} J(x,u) := \frac{1}{2}\sum_{k=1}^{n}\int_0^T |x_k(t) - x_k^d|^2 dt + \alpha \sum_{j=1}^{m}\int_0^T u_j(t)\,dt \\ &\text{s.t. } \frac{d}{dt}x_k = f_k(x,u),\, x_k(0) = x_k^0 \text{ for all k} \in \{1,...,\text{n}\} \text{ and } t \in (0,T) \end{aligned} \tag{1}$$

we present solution strategies in the following.

The formulation above is for continuous functions. For the calculations, we need to discretize the system as we can just do numerical calculations for a finite number of grid points in time. We have $t = ldt$ where $dt > 0$ is the time step, $l \in \{0, ..., N\}$ and $T = Ndt$. We discretize the ordinary differential equation $\frac{d}{dt}x_k = f_k(x,u)$ with the following explicit Euler scheme for each node k, see [5] for details about discretizing and solving ordinary differential equations numerically. This means that $\frac{d}{dt}x_k = f_k(x,u)$ is approximated by $\frac{x_k^{l+1} - x_k^l}{dt} = f_k\left(x^l, u^l\right)$ for each k where $x^l := \begin{pmatrix} x_1^l \\ \vdots \\ x_n^l \end{pmatrix}$, $u^l = \begin{pmatrix} u_1^l \\ \vdots \\ u_m^l \end{pmatrix}$ and x_k^l or u_j^l denotes the approximation to $x_k(ldt)$ or $u_j(ldt)$, respectively, for all $k \in \{1, ..., n\}$ and $j \in \{1, ..., m\}$. Then we obtain the following system of equations

$$\begin{aligned} x_k^1 &= x_k^0 + dt f_k\left(x^0, u^0\right) \\ x_k^2 &= x_k^1 + dt f_k\left(x^1, u^1\right) \\ &\vdots \\ x_k^N &= x_k^{N-1} + dt f_k\left(x^{N-1}, u^{N-1}\right) \end{aligned} \tag{2}$$

for every $k \in \{1, ..., n\}$. In the discretized framework, x_k is not a function any more but a vector $x_k := \begin{pmatrix} x_k^1 \\ \vdots \\ x_k^N \end{pmatrix} \in \mathbb{R}^N$ for all $k \in \{1, ..., n\}$ and $x := \begin{pmatrix} x_1 \\ \vdots \\ x_n \end{pmatrix} \in \mathbb{R}^{nN}$. Analogously, the vector $u_j := \begin{pmatrix} u_j^0 \\ \vdots \\ u_j^{N-1} \end{pmatrix} \in \mathbb{R}^N$ and $u := \begin{pmatrix} u_1 \\ \vdots \\ u_m \end{pmatrix} \in$

$\mathbb{R}^{mN}$. If we define

$$F_k(x,u) := -\begin{pmatrix} x_k^1 \\ \vdots \\ x_k^N \end{pmatrix} + \begin{pmatrix} x_k^0 \\ \vdots \\ x_k^{N-1} \end{pmatrix} + \begin{pmatrix} f_k\left(x^0, u^0\right) \\ \vdots \\ f_k\left(x^{N-1}, u^{N-1}\right) \end{pmatrix} dt \in \mathbb{R}^N,$$

then (2) can be written as $F_k(x,u) = 0$ for all $k \in \{1, ..., n\}$. Discretizing the integrals in of J with the Riemann sum, we obtain the following discretized optimization problem

$$\begin{aligned} &\min_{x,u} J(x,u) := \frac{1}{2}\sum_{k=1}^{n}\sum_{l=1}^{N} |x_k^l - x_k^d|^2 dt + \alpha \sum_{j=1}^{m}\sum_{l=0}^{N-1} u_j^l dt \\ &s.t.\ F(x,u) = 0,\ x^0 = x_0 \\ &u \in U_{ad} \end{aligned} \tag{3}$$

where $F(x,u) := \begin{pmatrix} F_1(x,u) \\ \vdots \\ F_n(x,u) \end{pmatrix} \in \mathbb{R}^{nN}$ and

$$U_{ad} := \left\{u \in \mathbb{R}^{mN} |\ 0 \leq u_j^l \leq 1 \text{ for all } j \in \{1, ..., m\} \text{ and } l \in \{0, ..., N-1\}\right\}$$

which means that all components of u are constraint according to the definition of the external stimuli.

One method to determine the external stimuli u which solve (3), a so called optimal solution to (3), is the Lagrange approach, see text books about mathematical optimization or Lagrange optimization like [8] for details. For this purpose, we have to formulate the Lagrange function which reads as follows

$$L(x,u,p) := \frac{1}{2}\sum_{k=1}^{n}\sum_{l=1}^{N} |x_k^l - x_k^d|^2 dt + \alpha \sum_{j=1}^{m}\sum_{l=0}^{N-1} u_j^l dt + \sum_{k=1}^{n} p_k^T F_k(x,u)\, dt$$

where $p := \begin{pmatrix} p_1 \\ \vdots \\ p_n \end{pmatrix} \in \mathbb{R}^{nN}$, $p_k := \begin{pmatrix} p_k^1 \\ \vdots \\ p_k^N \end{pmatrix} \in \mathbb{R}^N$, is a vector of so called Lagrange multipliers and $p_k^T F_k$ is the Euclidean scalar product of the vectors p_k and F_k for all $k \in \{1, ..., n\}$. Before we proceed, we introduce some symbols. We use the following scalar product for the following calculations. We have $(v,w) := \sum_{k=1}^{n}\sum_{l=1}^{N} v_k^l w_k^l dt$ for any $v, w \in \mathbb{R}^{nN}$. Analogously for vectors $v, w \in \mathbb{R}^{mN}$. The gradient of the Lagrange function L with respect to p is denoted by

$$\nabla_p L = \frac{1}{dt}\begin{pmatrix} \frac{\partial}{\partial p_1^1} L(x,u,p) \\ \frac{\partial}{\partial p_1^2} L(x,u,p) \\ \vdots \\ \frac{\partial}{\partial p_n^N} L(x,u,p) \end{pmatrix} \in \mathbb{R}^{nN},$$

see [1] for details about the connection between gradient and derivative. Analogously, the gradient of the Lagrange function L with respect to x is denoted by

$$\nabla_x L = \frac{1}{dt}\begin{pmatrix} \frac{\partial}{\partial x_1^1} L(x,u,p) \\ \frac{\partial}{\partial x_1^2} L(x,u,p) \\ \vdots \\ \frac{\partial}{\partial x_n^N} L(x,u,p) \end{pmatrix} \in \mathbb{R}^{nN}$$

and the gradient of the Lagrange function L with respect to u is denoted by

$$\nabla_u L = \frac{1}{dt}\begin{pmatrix} \frac{\partial}{\partial u_1^0} L(x,u,p) \\ \frac{\partial}{\partial u_1^1} L(x,u,p) \\ \vdots \\ \frac{\partial}{\partial u_m^{N-1}} L(x,u,p) \end{pmatrix} \in \mathbb{R}^{mN}.$$

Applying these formulas to the Lagrange function, we calculate the gradients for the Lagrange formalism. We have

$$\nabla_p L(x,u,p) = F(x,u).$$

Next, we have

$$\nabla_x L(x,u,p) = \begin{pmatrix} \nabla_{x_1} L(x,u,p) \\ \vdots \\ \nabla_{x_n} L(x,u,p) \end{pmatrix}$$

where

$$\nabla_{x_k} L(x,u,p) = \begin{pmatrix} x_k^1 - x_k^d - p_k^1 + p_k^2 + \sum_{i=1}^n p_i^2 \frac{\partial}{\partial x_k^1} f_i\left(x^1,u^1\right) dt \\ x_k^2 - x_k^d - p_k^2 + p_k^3 + \sum_{i=1}^n p_i^3 \frac{\partial}{\partial x_k^2} f_i\left(x^2,u^2\right) dt \\ \vdots \\ x_k^{N-1} - x_k^d - p_k^{N-1} + p_k^N + \sum_{i=1}^n p_i^N \frac{\partial}{\partial x_k^{N-1}} f_i\left(x^{N-1},u^{N-1}\right) dt \\ x_k^N - x_k^d - p_k^N \end{pmatrix} \in \mathbb{R}^N \tag{4}$$

for all $k \in \{1,...,n\}$. Notice that $\frac{\partial}{\partial x_k^1} f_i = ... = \frac{\partial}{\partial x_k^{N-1}} f_i$ holds for the derivatives in (4) if considered as functions from $\mathbb{R}^n \times \mathbb{R}^m$ to $\mathbb{R}$. Finally,

$$\nabla_u L(x,u,p) = \begin{pmatrix} \nabla_{u_1} L(x,u,p) \\ \vdots \\ \nabla_{u_m} L(x,u,p) \end{pmatrix}$$

where

$$\nabla_{u_j} L(x,u,p) = \begin{pmatrix} \alpha + \sum_{i=1}^n p_i^1 \frac{\partial}{\partial u_j^0} f_i\left(x^0,u^0\right) dt \\ \vdots \\ \alpha + \sum_{i=1}^n p_i^N \frac{\partial}{\partial u_j^{N-1}} f_i\left(x^{N-1},u^{N-1}\right) dt \end{pmatrix} \in \mathbb{R}^N \tag{5}$$

for all $j \in \{1,...,m\}$. Notice that $\frac{\partial}{\partial u_j^1} f_i = ... = \frac{\partial}{\partial u_j^{N-1}} f_i$ holds for the derivatives in (5) if considered as functions from $\mathbb{R}^n \times \mathbb{R}^m$ to $\mathbb{R}$.

Following the Lagrange approach to determine a solution u to (3) and thus the desired external stimuli, we have to solve the following system of equations, see [8, 1.5] for example,

$$\nabla_p L(x,u,p) = 0 \tag{6}$$

$$\nabla_x L(x,u,p) = 0 \tag{7}$$

$$\nabla_u L(x,u,p)^T (\tilde{u} - u) \geq 0 \text{ for all } \tilde{u} \in U_{ad}. \tag{8}$$

Equation (8) is a generalization of $\nabla_u L(x,u,p) = 0$ at a minimum. The need for the generalization comes from the fact that the vector u is not calculated in $\mathbb{R}^{mN}$, that means we can choose any value for u, but in U_{ad}, which means that there are restrictions on the choice of the values of u, see [8, Proposition 1.2].

Different strategies are available to solve the proposed optimization problem with the help of necessary equations like (6) to (8). We present three different algorithms that are used to calculate external stimuli for the desired switch by our software approach.

For a given u and as the initial value x_0 is given, Equation (6) can be solved as follows. We can calculate x_k^1 for all $k \in \{1,...,n\}$ from the first row of the corresponding $F_k(x,u)$. Then, we can calculate x_k^2 for all $k \in \{1,...,n\}$ from the second row of the corresponding $F_k(x,u)$. This procedure can be repeated, going forwards through all the rows of the corresponding $F_k(x,u)$ until x_k^N for all $k \in \{1,...,n\}$ is calculated from the last row of the corresponding $F_k(x,u)$. Analogously, for a given u and x, (7) can be calculated, however, starting from the last row of $\nabla_{x_k} L(x,u,p)$ to determine the corresponding p_k^N for each $k \in \{1,...,n\}$ and then going backwards through all the rows of $\nabla_{x_k} L(x,u,p)$ until the corresponding p_k^1 can be calculated from the first row of $\nabla_{x_k} L(x,u,p)$ for all $k \in \{1,...,n\}$. The vector $\nabla_u L(x,u,p)$ can be assembled for given u, x and p. Any vector $u \in U_{ad}$ which fulfills (8) for a given x and p is a minimizer of the Lagrange function $L(x,u,p)$. There are various methods to calculate u such that (8) is fulfilled and simultaneously (6) and (7) are fulfilled, see [2, 3, 8]. We choose a projected gradient method, see Algorithm 1 to calculate a solution u sought. For this purpose, we need to define the projection $Pr : \mathbb{R}^m \to \mathbb{R}^m$ of a vector u that is

given by

$$Pr(u) = \begin{cases} u_j^l & \text{if } 0 < u_j^l < 1 \\ 1 & \text{if } u_j^l \geq 1 \\ 0 & \text{if } u_j^l \leq 0. \end{cases}$$

Before we present the projected gradient method which illustrates the algorithmic method how to minimize the target functional in a very direct way, we give the following remark.

Remark 1. The gradient $\nabla_u L(x, u, p)$ is associated with the gradient $\nabla \hat{J}(u)$ of the reduced target functional $\hat{J}(u) := J(x(u), u)$, see [8, Proof of Theorem 1.17]. Actually, we have that for variations in u, the function $x(u)$ also varies as x and u are connected via (2). Furthermore, for a given u, the function $x(u)$ can be calculated by $F(x, u) = 0$ as discussed above. The gradient $\nabla \hat{J}(u)$ can be used for any optimization method based on a steepest descent in order to find an optimal control for (3), like for a nonlinear conjugated gradient (NCG) method or a Broyden-Fletcher–Goldfarb-Shanno (BFGS) method. With these methods one can extend the algorithmic part of this work with respect to the calculation of an optimal solution for (3).

Algorithm 1 Projected gradient method

1. Choose $\sigma \in (0,1)$, ${}^0s = 1$, $\beta_1 \in (0,1)$, $\beta_2 > 1$, $\epsilon > 0$, $i = 0$, ${}^0u \in U_{ad}$
2. Calculate ${}^0x$ from (6) for ${}^0u$ and ${}^0p$ from (7) for ${}^0x$ and ${}^0u$
3. While $\left({}^iu - Pr\left({}^iu - \nabla_u L\left({}^ix, {}^iu, {}^ip\right)\right)\right)^T \left({}^iu - Pr\left({}^iu - \nabla_u L\left({}^ix, {}^iu, {}^ip\right)\right)\right) > \epsilon$
   Determine ${}^{i+1}s = {}^is \cdot \max\left\{\beta_1^\ell \mid \ell = 0, 1, ...\right\}$ with

$$\hat{J}\left(Pr\left({}^iu - {}^{i+1}s\nabla_u L\left({}^ix, {}^iu, {}^ip\right)\right)\right) \leq \hat{J}\left({}^iu\right) - \sigma \nabla_u L\left({}^ix, {}^iu, {}^ip\right)^T \left({}^iu - Pr\left({}^iu - {}^{i+1}s\nabla_u L\left({}^ix, {}^iu, {}^ip\right)\right)\right)$$

   Set ${}^{i+1}u = Pr\left({}^iu - {}^{i+1}s\nabla_u L\left({}^ix, {}^iu, {}^ip\right)\right)$
   Calculate ${}^{i+1}x$ from (6) for ${}^{i+1}u$ and ${}^{i+1}p$ from (7) for ${}^{i+1}x$ and ${}^{i+1}u$
   Set ${}^{i+1}s = \beta_2 {}^{i+1}s$
   Set $i = i + 1$
   End

We give an explanation of Algorithms 1. Algorithm 1 iteratively determines a solution to the Equations (6) - (8). The notation ${}^ix \in \mathbb{R}^n$, ${}^iu \in \mathbb{R}^m$ and ${}^ip \in \mathbb{R}^n$ means that these vectors contain the values for the corresponding vectors x, u and p at the corresponding entry after the i-th iteration of Algorithm 1. The more iterations Algorithm 1 performs, the better a solution is calculated by Algorithm 1. For this purpose, the parameter ϵ is a measure how close the numerical solution of Algorithm 1 is to an analytical solution to the Equations (6) - (8). The smaller ϵ is, the closer the numerical solution is to an analytical one but the calculation time increases. Typically, the parameter ϵ ranges between 10^{-3} to 10^{-9}. We recommend $\epsilon = 10^{-4}$ to obtain useful results for the external stimuli framework. The step size ${}^is$ is determined such that it is associated with reducing $\hat{J}$. The gradient $\nabla_u L(x, u, p) \in \mathbb{R}^{mN}$ points out in the direction of the steepest ascent of $L(x, u, p)$ or $\hat{J}(u)$, respectively and thus $-\nabla_u L(x, u, p)$ points out in the direction of the steepest descent. The projection of ${}^iu - {}^{i+1}s\nabla_u L\left({}^ix, {}^iu, {}^ip\right)$ on U_{ad} is needed to ensure that each iterate is in U_{ad} and thus the output of Algorithm 1 is in U_{ad}.

This formulation of a projected gradient method noted in Algorithm 1 has the advantage that the step size stays in the size of magnitude from the last iteration. According to our experience the step size stays in the same size of magnitude over several iterations. Therefore, in such a case, our formulation has the advantage that the step size does not have to be found in each iteration from the scratch and thus can save calculation time. Additionally, the step size is adaptively found from an initial guess and varied by slightly enlarging it after each iteration in the case that a greater step size might be suitable and thus generate a greater descent of the target functional. From our experience, we recommend $\sigma = 0.01$, $\beta_1 = 0.1$ and $\beta_2 = 1.1$ for a fast convergence of Algorithm 1.

We provide another algorithm that is different form the projected gradient method for the case that the projected gradient method converges slowly for a special setting consisting of a certain network equipped with external stimuli. For this case an alternative method is implemented and provided with this work which might perform better in such a case. For this purpose we characterize a solution of an optimal control system (1) with the Pontryagin maximum principle (PMP), see for example [7, 2.4]. Before we discuss it further, we define the Hamiltonian for our problem as

follows $H : \mathbb{R} \times \mathbb{R}^n \times \mathbb{R}^m \times \mathbb{R}^n \to \mathbb{R}$, $(t, x, u, p) \mapsto H(t, x, u, p)$ where

$$H(t, x, u, p) := \frac{1}{2} \sum_{k=1}^{n} \left(x_k - x_k^d\right)^2 + \alpha \sum_{j=1}^{m} u_j + \sum_{k=1}^{n} p_k f_k(x, u).$$

The PMP says that it holds

$$H(t, \bar{x}(t), \bar{u}(t), \bar{p}(t)) = \min_{w \in [0,1]^m} H(t, \bar{x}(t), w, \bar{p}(t)) \tag{9}$$

for almost every $t \in [0, T]$ where the functions $(\bar{x}, \bar{u})$ fulfill (1). Furthermore, the adjoint $\bar{p}$ is given componentwise by the following adjoint equation

$$\frac{d\bar{p}_k}{dt} = -\left(\bar{x}_k - x_k^d\right) - \sum_{i=1}^{n} \bar{p}_i \frac{\partial}{\partial x_k} f_i(\bar{x}, \bar{u}) \tag{10}$$

with $\bar{p}_k(T) = 0$ for all $k \in \{1, ..., n\}$ where $\frac{\partial}{\partial x_k} f_i(\bar{x}, \bar{u}) := \frac{\partial}{\partial x_k} f_i(x, u)|_{(x,u)=(\bar{x},\bar{u})}$ that means the partial derivative of the i-th component of f with respect to the k-th component of x evaluated at $(\bar{x}, \bar{u})$. If one has any solution (x^*, u^*) to the constraint of (1), then minimizing $H(t, x^*(t), w, p^*(t))$ over $w \in [0,1]^m$ is related to minimizing the target functional of (1) as [7, 2.4.2] indicates where p^* is a solution to (10) for x^* and u^* instead of $\bar{x}$ and $\bar{u}$, respectively. This connection is made use of in the sequential quadratic Hamiltonian method. For this method, the augmented Hamiltonian is crucial which is given by $K_\epsilon : \mathbb{R} \times \mathbb{R}^n \times \mathbb{R}^m \times \mathbb{R}^m \times \mathbb{R}^n \to \mathbb{R}$, $(t, x, u, v, p) \mapsto K_\epsilon(t, x, u, v, p)$ with $K_\epsilon(t, x, u, v, p) := H(t, x, u, p) + \epsilon \sum_{i=j}^{m} (u_j - v_j)^2$. There are a number of different variants to implement Pontryagin's maximum principle. We use the following where according to our experience this method converges faster to appropriate results than the projected gradient method.

Algorithm 2 Sequential quadratic Hamiltonian method

1. Choose $\epsilon > 0$, $\kappa > 0$, $\sigma > 1$, $\zeta \in (0, 1)$, $\eta \in (0, \infty)$, u^0, compute x^0 with the constraint of (1) for $u \leftarrow u^0$ and p^0 from (10) for $\bar{x} \leftarrow x^0$ and $\bar{u} \leftarrow u^0$, set $\ell \leftarrow 0$
2. Choose u such that
$$K_\epsilon\left(t, x^\ell(t), u(t), u^\ell(t), p^\ell(t)\right) \leq K_\epsilon\left(t, x^\ell(t), w, u^\ell(t), p^\ell(t)\right)$$
for all $w \in [0,1]^m$ and all grid points $t \in [0, T]$
3. Calculate x from the constraint of (1) for u and $\delta := \|u - u^\ell\|^2 := \int_0^T \left(u(t) - u^\ell(t)\right)^2 dt$
4. If $J(x, u) - J\left(x^\ell, u^\ell\right) > -\eta\delta$: Choose $\epsilon \leftarrow \sigma\epsilon$
Else: Choose $\epsilon \leftarrow \zeta\epsilon$, $x^{\ell+1} \leftarrow x$, $u^{\ell+1} \leftarrow u$, calculate $p^{\ell+1}$ from (10) for $\bar{x} \leftarrow x^{\ell+1}$ and $\bar{u} \leftarrow u^{\ell+1}$, $\ell \leftarrow \ell + 1$
5. If $\delta < \kappa$: STOP and return u^ℓ
Else go to 2.

Algorithm 2 works as follows. First, one chooses an initial guess for ϵ, recommended $\epsilon = 0.1$, which weights the deviation from the current u^ℓ to the update, a tolerance κ for the convergence criterion, σ for enlarging ϵ, ζ for diminishing ϵ, η for the comparison of the value of the target functional for the current control u^ℓ and its update. We recommend $\sigma = 50$, $\zeta = 0.15$ and $\eta = 10^{-12}$ and $\kappa = 10^{-6}$. Furthermore, one chooses an initial guess for the control u^0. With that guess, one computes x^0 with the constraint of (1) and u^0 instead of u as well as p^0 with (10) for x^0 instead of $\bar{x}$, we write $\bar{x} \leftarrow x^0$ and u^0 instead of $\bar{u}$, we write $\bar{u} \leftarrow u^0$. Secondly, one calculates the minimum of $K_\epsilon\left(t, x^\ell, w, u^\ell, p^\ell\right)$ for every grid point t which is in our case given by $0 = \frac{\partial}{\partial u_j} K_\epsilon\left(t, x^\ell, u, u^\ell, p^\ell\right)$, where $\frac{\partial}{\partial u_j} K_\epsilon\left(t, x^\ell, u, u^\ell, p^\ell\right)$ is the partial derivative of K_ϵ with respect of the j-th component of the control u in the third argument of K_ϵ. Resolving that considering the upper and lower bound of u, one obtains for the update of each control $j \in \{1, ..., m\}$ the following

$$u_j(t) = \max\left(0, \min\left(-\frac{\alpha + \sum_{i=1}^{n} p_i^\ell(t) \frac{\partial f_i\left(x^\ell, u\right)}{\partial u_j}}{2\epsilon} + u_j^\ell(t), 1\right)\right) \tag{11}$$

for each grid point $t \in [0, T]$ where p_i^ℓ is the i-th component of p^ℓ. We remark that $\frac{\partial f_i\left(x^\ell, u\right)}{\partial u_j}$, the partial derivative of the i-th component of f with respect to the j-th component of the control u, does actually not depend on u_j in

our case and thus 11 holds for any case where $\frac{\partial f_i(x^\ell,u)}{\partial u_j}$ does not depend on u_j. In the next step, we calculate x from the constraint of (1) for u, $x(0) = x_0$, the norm square $\|u - u^\ell\|^2$ with the corresponding Riemann sum and set $\delta := \|u - u^\ell\|^2$. In the forth step, we check if the new update on u provides a smaller value of the target functional by at least $-\eta\delta$. If not, we enlarge ϵ, else we accept the update for the state and control, diminish ϵ and calculate p^ℓ from (10) with $p^\ell(T) = 0$, with the new x^ℓ, $\bar{x} \leftarrow x^\ell$ and u^k, $\bar{u} \leftarrow u^\ell$. If at a certain iteration δ is less than the tolerance κ, we stop and return the last iterate u^ℓ which has provided a descent of the target functional by at least $-\eta\delta$.

When solving (1) or (3), respectively, in order to find a set of external stimuli which causes the switch from the initial state x_0 to the desired state x_d, it may happen because of the highly non-linear constraint F or f_k, respectively, that there is a local optimum for the target functional at which there is a node that does not equal its desired state at the final time. This can happen, because Algorithm 1 and Algorithm 2 are so called local optimization methods and therefore they only determine their result with respect to necessary optimality conditions (8) or (9), respectively. However, not all optima in this sense contain a switch of steady states. Thus if the initial value x_0 is close to such a local minimum, it may happen that our utilized local optimization method like projected gradient method or sequential quadratic Hamiltonian method, converges to such a mentioned local optimum. Then, the corresponding external stimuli are optimal in the sense of fulfilling the necessary optimality conditions (8) or (9) but may not cause the desired switch. Moreover, varying the initial guess for the external stimuli u in Algorithm 1 or Algorithm 2 can cause convergence to different local optima and thus, considering even just the local minima containing a desired switch, can result in different sets of active external stimuli.

In order to prevent converging to a local optimum which does not include a switch of the network's state, one has to elaborate a way how to find an initial guess of external stimuli where the network is close to an optimum containing that the corresponding state equals the desired state at the final time. For this purpose, we propose the following method, which we can perform before a local optimization method in order to come close to a desired solution. We recall that $m \in \mathbb{N}$ is the number of external stimuli, $n \in \mathbb{N}$ the number of nodes and T the time horizon. The method considers all the elements g of the power set $\mathcal{P}$ of the set $\{1, ..., m\}$ up to a cardinality $|g| \leq \text{maxNum}$ where $\text{maxNum} \in \{1, ..., m\}$. Then for each g, we define the following controls

$$u_j(\eta T) := \begin{cases} 1 & \text{for } 0 \leq t \leq \eta T \\ 0 & \text{else,} \end{cases}$$

$0 < \eta < 1$ for all $j \in p$. For $j \in \{1, ..., m\} \setminus p$, we set $u_j(\eta T) = 0$ for all $0 \leq t \leq T$ and define

$$u(\eta T) := \begin{pmatrix} u_1(\eta T) \\ \vdots \\ u_m(\eta T) \end{pmatrix}. \tag{12}$$

That means we pick systematically combinations of subsets of the index set $\{1, ..., m\}$ and apply the corresponding controls for a certain period of time starting from $t = 0$ until all the controls are switched off after ηT time units. If the state $x(T)$ of the network at the final time T equals the desired state x_d in each component up to a tolerance $\text{tol} > 0$, then we figure out if the switch can be achieved with a shorter period of the controls' application than ηT. For this purpose, we take $u(\tau^l \eta T)$ with the corresponding state calculated by $\frac{d}{dt}x_k = f(x, u(\tau^l \eta T))$ for all $k \in \{1, ..., n\}$ or $F(x, u(\tau^l \eta T))$, respectively, with $0 < \tau < 1$, $l \in \mathbb{N}_0$ and enlarge l until there is a state of one node, x_k, $k \in \{1, ..., n\}$, for which we have $|x_k(T) - (x_d)_k| \geq \text{tol}$. We note the smallest number where the switch does not happen any more $\tilde{l} \in \mathbb{N}$. Then we take the duration of application $\tau^{\tilde{l}-1}\eta T$ such that we define the control $u\left(\tau^{\tilde{l}-1}\eta T\right)$ as the output of the combinatorial method. The method is given as follows.

Algorithm 3 Combinatorial method

1. Choose maxNum $\in \{1, ..., m\}$, $0 < \text{tol} < 1$, $0 < \eta < 1$, $0 < \tau < 1$,
2. Choose all the elements $g \in P \subseteq \mathcal{P}$ of the power set $\mathcal{P}$ of $\{1, ..., m\}$ such that the cardinality $|g| \leq$ maxNum
3. For all $g \in P$
   - Set $u = u(\eta T)$ according to (12)
   - Calculate x with u from $\frac{d}{dt} x_k = f(x, u)$ for all $k \in \{1, ..., n\}$defined in (1) or $F(x, u) = 0$ defined in (3), respectively
   - If $|x_k(T) - (x_d)_k| < \text{tol}$ for all $k \in \{1, ..., n\}$, return $u\left(\tau^{\tilde{l}-1}\eta T\right)$ where $\tilde{l} \in \mathbb{N}$ is the smallest number such that there is one $k \in \{1, ..., n\}$ with $|x_k(T) - (x_d)_k| \geq \text{tol}$ with $\frac{d}{dt} x_k = f\left(x, u\left(\tau^{\tilde{l}}\eta T\right)\right)$ for all $k \in \{1, ..., n\}$ or $F\left(x, u\left(\tau^{\tilde{l}}\eta T\right)\right) = 0$, respectively

We remark that the time horizon T used in Algorithm 3, Algorithm 2 and Algorithm 1 is supposed to be at least that large as in the steady state analysis in which one figures out the steady states of the considered network in order to ensure that there is enough time for the network to relax into the desired state after the external stimuli are switched off. This is reasonable because the oscillations induced by the switch on and switch off of the external stimuli can decay and the network can relax to the desired state x_d sufficiently much such that $x_k(T)$ is close to $(x_d)_k$ within the tolerance tol for all $k \in \{1, ..., n\}$. Also the step size dt which is supposed to be at most that size as the one used in the steady state analysis in order to ensure a stable numerical solution of the underlying differential equations assuming that an explicit solver for the steady state analysis is used.

The advantage of Algorithm 3 is that it checks directly if the desired switch is achieved with the chosen set of external stimuli. However, the disadvantage is that it needs exponential time with respect to the number of possible external stimuli. In contrast, Algorithm 1 or Algorithm 2 are linear in time with respect to the number of possible external stimuli and at most quadratic with respect to the total number of nodes and edges as one has to calculate the Jacobi matrix of f, see for example (4). Therefore one can combine these two methods as follows in order to determine a set of external stimuli supporting the desired switch. Even for a large number of possible external stimuli, the number of combinations is not too big if maxNum is small. If then Algorithm 3 returns a set of external stimuli causing the desired switch, one can use this result as an initial guess for the external stimuli for Algorithm 1 or Algorithm 2 in order to find more external stimuli supporting the desired switch which can be affected by the weight α in J of (1). Roughly spoken, the less α is, the more different external stimuli will be found which are different from constant zero function and the bigger α is, the less different controls are returned being different from zero, but the more important these external stimuli are for the switch. Supporting the desired switch means that the network is steered faster to the desired state. That can be useful for dynamical reasons when time matters that means a switch in an experiment is supposed to be accelerated or to enlarge the number of external stimuli a bit to choose that ones that can be implemented in an experimental set up in the most easy way. Further Algorithm 3 returns the first combination of external stimuli that it finds. However, there might be a combination that is more effective for the switch that is more likely to be found from Algorithm 1 or Algorithm 2, especially for big α. For this reason it is worth to additionally start one of these algorithms with the result of Algorithm 3 as an initial guess.

2 A case study to show the basic application of the proposed optimization framework

In this section, we demonstrate how to analyze a regulatory network which includes interactions with external stimuli using our optimization approach. The aim of this section is to demonstrate how in principle use the approach for network analysis with respect to induce a switch of steady states with external stimuli and to get familiar with the interpretation of the results of the method. For our numerical examples, we use the regulatory network from [10, Figure 5], Alternative Th network. The schematic of this network is given in Figure A where IFN-γ is node 1, CSIF is node 2, IL-2 is node 3 and IL-4 is node 4. Though this network is really small (4 nodes) it includes cross connectivity typical of biological systems, for instance also in the TNF network [11], in cardiomyocytes [4] or proliferation vs. apoptosis in cells [12, 6].

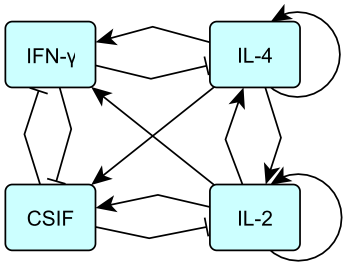

Figure A: The schematic of our example network, see [10, Figure 5].

In our first example, we analyze the transit of one steady state to another for a given set of external stimuli. We consider the corresponding equations containing external stimuli, see Section 2, Equation (2) of the main manuscript, with u_1 activating node 1, u_2 activating node 4 and u_3 activating node 2, as follows

$$\begin{aligned}
\frac{d}{dt}x_1 &= \frac{-\mathrm{e}^5 + \mathrm{e}^{-10\left(\frac{3}{2}\left(\frac{x_3+x_4}{1+x_3+x_4}\right)\left(1-\frac{11}{10}\frac{10x_2}{1+10x_2}\right)-0.5\right)}}{\left(1-\mathrm{e}^5\right)\left(1+\mathrm{e}^{-10\left(\frac{3}{2}\left(\frac{x_3+x_4}{1+x_3+x_4}\right)\left(1-\frac{11}{10}\frac{10x_2}{1+10x_2}\right)-0.5\right)}\right)} - x_1 + u_1\left(1-x_1\right)\\
\frac{d}{dt}x_2 &= \frac{-\mathrm{e}^5 + \mathrm{e}^{-10\left(\frac{3}{2}\left(\frac{x_3+x_4}{1+x_3+x_4}\right)\left(1-\frac{11}{10}\frac{10x_1}{1+10x_1}\right)-0.5\right)}}{\left(1-\mathrm{e}^5\right)\left(1+\mathrm{e}^{-10\left(\frac{3}{2}\left(\frac{x_3+x_4}{1+x_3+x_4}\right)\left(1-\frac{11}{10}\frac{10x_1}{1+10x_1}\right)-0.5\right)}\right)} - x_2 + u_3\left(1-x_2\right)\\
\frac{d}{dt}x_3 &= \frac{-\mathrm{e}^5 + \mathrm{e}^{-10\left(\frac{3}{2}\left(\frac{x_3+x_4}{1+x_3+x_4}\right)\left(1-\frac{11}{10}\frac{10x_2}{1+10x_2}\right)-0.5\right)}}{\left(1-\mathrm{e}^5\right)\left(1+\mathrm{e}^{-10\left(\frac{3}{2}\left(\frac{x_3+x_4}{1+x_3+x_4}\right)\left(1-\frac{11}{10}\frac{10x_2}{1+10x_2}\right)-0.5\right)}\right)} - x_3\\
\frac{d}{dt}x_4 &= \frac{-\mathrm{e}^5 + \mathrm{e}^{-10\left(\frac{3}{2}\left(\frac{x_3+x_4}{1+x_3+x_4}\right)\left(1-\frac{11}{10}\frac{10x_1}{1+10x_1}\right)-0.5\right)}}{\left(1-\mathrm{e}^5\right)\left(1+\mathrm{e}^{-10\left(\frac{3}{2}\left(\frac{x_3+x_4}{1+x_3+x_4}\right)\left(1-\frac{11}{10}\frac{10x_1}{1+10x_1}\right)-0.5\right)}\right)} - x_4 + u_2\left(1-x_4\right)
\end{aligned} \tag{13}$$

where we choose $h = 10$, $\alpha_k = 1$, $\beta_k = 10$ and $\gamma_k = 1$ for all $k \in \{1,2,3,4\}$. This choice applies for the rest of this section. We use (2) from the main manuscript and insert die activating and inhibiting nodes of the network depicted in Figure A. The external stimuli are also implemented according to the formalism explained with (2) from the main manuscript. Equation (2) from the main manuscript can also be obtained with the provided software by inputting the graph depicted in Figure A. In our example, we switch the regulatory network (13) from the steady state $x_0 = \begin{pmatrix} 0 & 0 & 0 & 0 \end{pmatrix}$ to the steady state $\begin{pmatrix} 0.8870 & 5.6662 \cdot 10^{-4} & 0.8870 & 5.6662 \cdot 10^{-4} \end{pmatrix}$. We remark that we round the numbers here in the work for notation reasons and that the symmetry of the connection of the interaction graph is reflected in the symmetry of the values in each steady state. Utilizing Algorithm 1 with $\alpha = 0.1$, $T = 20$, $dt = 0.1$, $\epsilon = 10^{-6}$ and ${}^0u$ with constant zero values, we obtain the results shown in Figure B and Figure C.

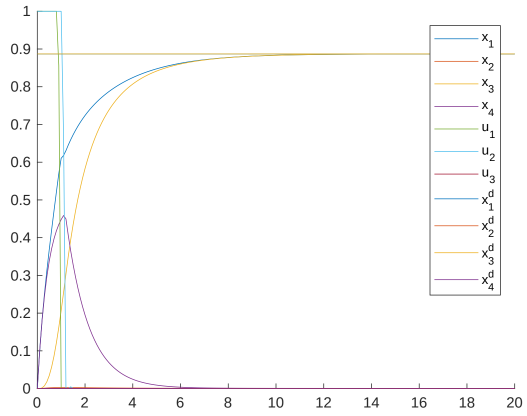

Figure B: The time is plotted on the abscissa and the activation level is plotted on the ordinate. Transition from the initial steady state to the desired steady state by the external stimuli u_1, u_2 calculated by Algorithm 1.

In Figure B, we see that the regulatory network is steered to the desired steady state by external stimuli u_1 and u_2. For this purpose, only a short application is necessary. Furthermore, we see that the stimulus u_3 is not needed for this transition. We remark that although we desire the activation level x_4 to be low, it is intermediately activated by u_2 in order to steer the whole regulatory network into the desired steady state. When u_2 is switched off, the activation level x_4 immediately declines.

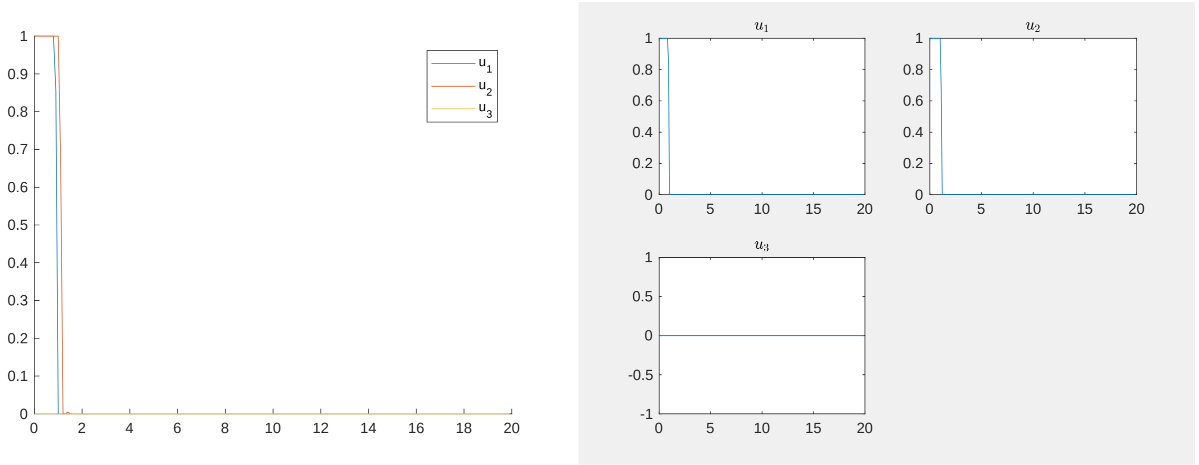

Figure C: The time is plotted on the abscissa and the activation level is plotted on the ordinate. External stimuli u_1, u_2 and u_3 in detail.

Even when not knowing the exact parameters of a system modeled by our equations, we can use Figure C as a qualitative information, as the model in [10] is intended to in the lack of the exact values for the model parameters. The qualitative information is that the external stimuli u_1 and u_2 are essential for the desired transition. Because of the qualitative character, the duration of application proposed by Algorithm 1 is hardly able to be recovered in a real experiment in general. However, in Figure D, we apply u_1 and u_2 much longer than proposed by Algorithm 1 while u_3 is switched off the whole time. Thus, we see that the model is robust with respect to a duration of application of the external stimuli that is longer than proposed by the calculation of Algorithm 1 because we obtain the same switch. After the external stimuli are switched off, the regulatory network relaxes into the desired steady state. We tell that the activation level x_4 of node 4 cannot exceed 0.5 although u_2 is maximum active.

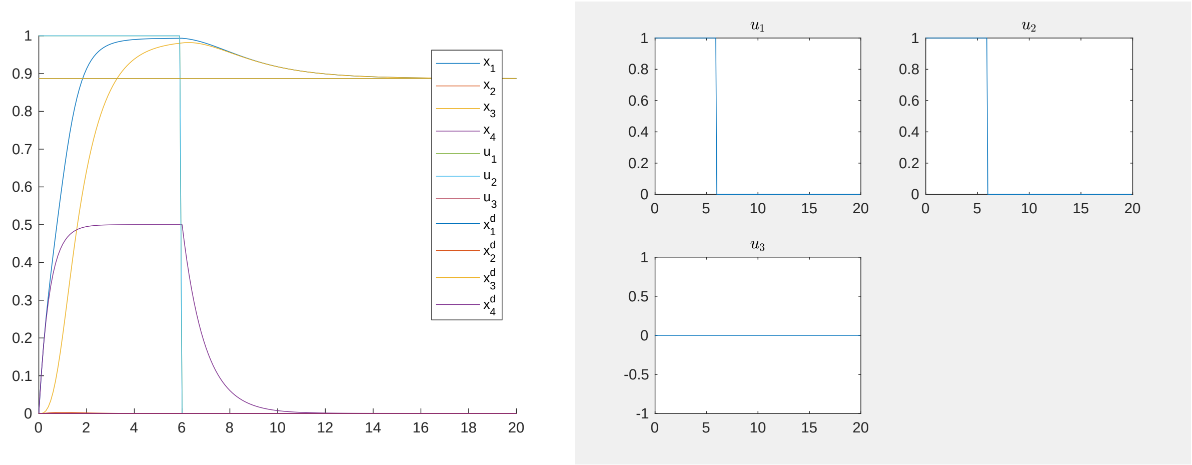

Figure D: The time is plotted on the abscissa and the activation level is plotted on the ordinate. Transition from the initial steady state to the desired steady state by the external stimuli u_1 and u_2 applied longer than proposed by Algorithm 1.

We remark that there might exist several selections of single external stimuli and their duration of application which steer the regulatory network from one steady state to another. Algorithm 1 calculates optimal external stimuli where optimal is meant in the sense of being a (local) solution to (3). The presented framework also offers the possibility to find external stimuli for the desired switch by trial and error methods, like Algorithm 3. For this purpose, one can set external stimuli and solve (2) to see if this choice has the desired effect on the regulatory network. Of course, this choice might have a bigger value of the target functional of (3) and is not optimal in this sense although it performs the desired switch. However, we focus on finding a selection of external stimuli which performs a desired switch. Solving (1) or (3) can be interpreted as an additional tool of systematic inspiration for us to find such a selection.

Algorithm 1 or Algorithm 2 in combination with the presented optimization approach provides a tool for systematically figuring out what external stimuli are essential to switch the regulatory network between different steady states. This is very useful for network analysis if the modeled system contains a lot of possibilities for activation or inhibition through external stimuli because the number of possible combinations of different external stimuli grows exponentially with the number of external stimuli. Thus a trial and error method to determine a set of essential external stimuli might become cumbersome, see the following example. In the following, we demonstrate the principle procedure with an easy example. The biological relevance of the optimal solution is then that it represents the pharmacological intervention, for instance, that induces the desired switch in the experiment if the model is sufficiently appropriate for the experiment. This example also demonstrates how from the amount of all external stimuli that support a desired switch the most effective ones are extracted which is also an important issue in pharmacological problems.

We equip a certain set of nodes with an activating or inhibiting external stimulus in order to determine a set of essential external stimuli. If a system is modeled with a regulatory network, one can generate candidate nodes for which one has to develop drugs for activation or inhibition such that one achieves the desired switch in the system.

As an illustrative example, we use the following

$$
\begin{aligned}
\frac{d}{dt}x_1 &= \frac{-\mathrm{e}^5 + \mathrm{e}^{-10\left(\frac{3}{2}\left(\frac{x_3+x_4}{1+x_3+x_4}\right)\left(1-\frac{11}{10}\frac{10x_2}{1+10x_2}\right)-0.5\right)}}{\left(1-\mathrm{e}^5\right)\left(1+\mathrm{e}^{-10\left(\frac{3}{2}\left(\frac{x_3+x_4}{1+x_3+x_4}\right)\left(1-\frac{11}{10}\frac{10x_2}{1+10x_2}\right)-0.5\right)}\right)} - x_1 + u_1\left(1-x_1\right) - u_2 x_1 \\
\frac{d}{dt}x_2 &= \frac{-\mathrm{e}^5 + \mathrm{e}^{-10\left(\frac{3}{2}\left(\frac{x_3+x_4}{1+x_3+x_4}\right)\left(1-\frac{11}{10}\frac{10x_1}{1+10x_1}\right)-0.5\right)}}{\left(1-\mathrm{e}^5\right)\left(1+\mathrm{e}^{-10\left(\frac{3}{2}\left(\frac{x_3+x_4}{1+x_3+x_4}\right)\left(1-\frac{11}{10}\frac{10x_1}{1+10x_1}\right)-0.5\right)}\right)} - x_2 + u_3\left(1-x_2\right) - u_4 x_2 \\
\frac{d}{dt}x_3 &= \frac{-\mathrm{e}^5 + \mathrm{e}^{-10\left(\frac{3}{2}\left(\frac{x_3+x_4}{1+x_3+x_4}\right)\left(1-\frac{11}{10}\frac{10x_2}{1+10x_2}\right)-0.5\right)}}{\left(1-\mathrm{e}^5\right)\left(1+\mathrm{e}^{-10\left(\frac{3}{2}\left(\frac{x_3+x_4}{1+x_3+x_4}\right)\left(1-\frac{11}{10}\frac{10x_2}{1+10x_2}\right)-0.5\right)}\right)} - x_3 + u_5\left(1-x_3\right) - u_6 x_3 \\
\frac{d}{dt}x_4 &= \frac{-\mathrm{e}^5 + \mathrm{e}^{-10\left(\frac{3}{2}\left(\frac{x_3+x_4}{1+x_3+x_4}\right)\left(1-\frac{11}{10}\frac{10x_1}{1+10x_1}\right)-0.5\right)}}{\left(1-\mathrm{e}^5\right)\left(1+\mathrm{e}^{-10\left(\frac{3}{2}\left(\frac{x_3+x_4}{1+x_3+x_4}\right)\left(1-\frac{11}{10}\frac{10x_1}{1+10x_1}\right)-0.5\right)}\right)} - x_4 + u_7\left(1-x_4\right) - u_8 x_4
\end{aligned}
\tag{14}
$$

where each node is equipped with an activating and an inhibiting external stimulus. We desire a switch from the steady state $x_0 = \begin{pmatrix} 0.8870 & 5.6662 \cdot 10^{-4} & 0.8870 & 5.6662 \cdot 10^{-4} \end{pmatrix}$ to the steady state $\begin{pmatrix} 5.6662 \cdot 10^{-4} & 0.8870 & 5.6662 \cdot 10^{-4} & 0.8870 \end{pmatrix}$. The results from Algorithm 1 with $\alpha = 0.1$, $T = 20$, $dt = 0.1$, $\epsilon = 10^{-6}$ and ${}^{0}u$ with constant zero values can be seen in Figure E. This selection of external stimuli is able to cause the desired switch of steady states, see Figure E.

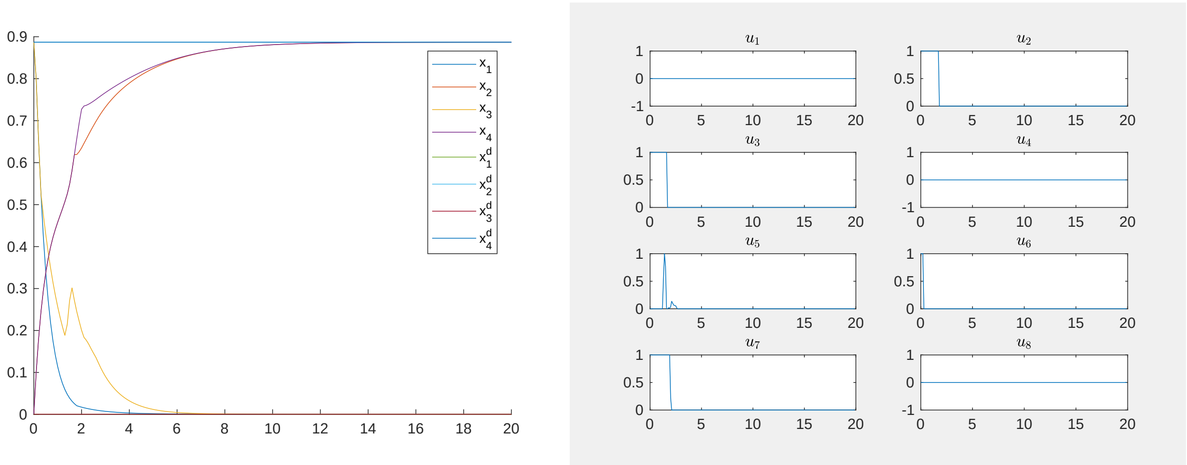

Figure E: The time is plotted on the abscissa and the activation level is plotted on the ordinate. An optimal selection of external stimuli performing the desired switch for $\alpha = 0.1$ and the time course of the nodes' activation level for the corresponding external stimuli.

For the same setting but with $\alpha = 1.1$ instead, we get a smaller number of non-zero external stimuli, as u_2 and u_6 are constant zero functions now, see Figure F. Comparing Figure E and Figure F, we see that the desired switch is performed faster in Figure E as more external stimuli are involved.

A further important information that can be obtained by performing the same experiment several times with increasing α is which external stimuli become a constant zero function. The external stimuli that remain are the most efficient ones for the switch because their input gives the biggest gain. The same can be interpreted as follows. All the external stimuli that are not the constant zero function are promising to focus on in order to induce a desired switch in the experiment.

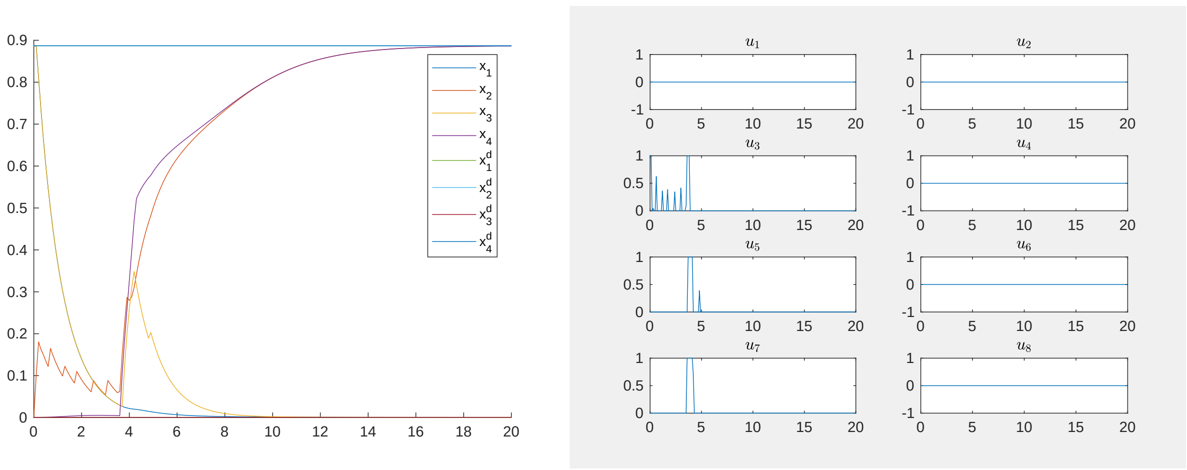

Figure F: The time is plotted on the abscissa and the activation level is plotted on the ordinate. An optimal selection of external stimuli performing the desired switch for $\alpha = 1.1$.

From Figure F, we see that external stimulus u_3 is applied longer than u_5 and u_7. Applying just u_3 and u_5 or u_3 and u_7 also performs the desired switch, see Figure G.

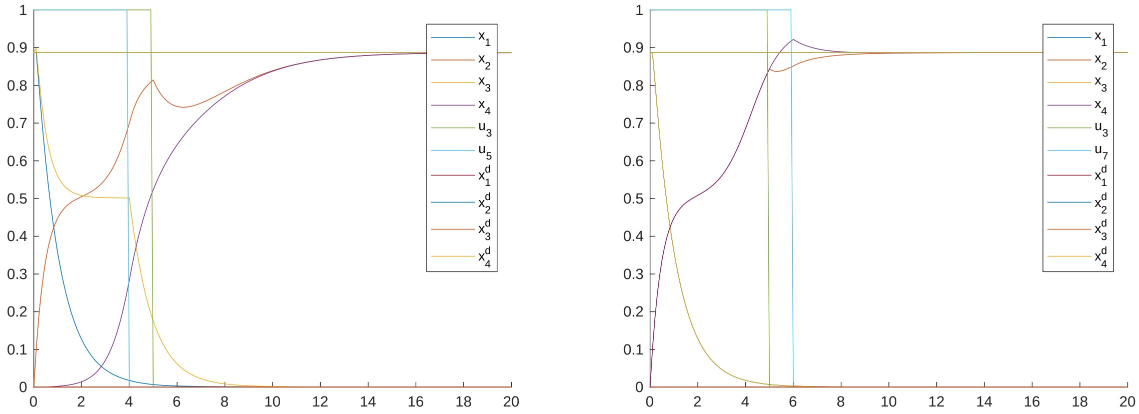

Figure G: The time is plotted on the abscissa and the activation level is plotted on the ordinate. Two suitable selections of external stimuli being able to perform the desired switch.

In order to obtain the desired switch, we observe a robustness with respect to the duration of application and the time curves of the external stimuli comparing the external stimuli in Figure G with the optimal ones in Figure F because both variants induce the desired switch. In other words, the exact time curve of the applied external stimuli is not decisive for the desired switch.

Applying just u_3 or u_5 and u_7 as in Figure H does not have the desired effect on the network. This illustrates the fact that there exist effects in that kind of networks such that a big duration and strength of a single external stimulus is not able to trigger a desired switch but rather the coordinated occurrence of different external stimuli is crucial for the desired switch. Summarizing this experiment, from all eight external stimuli, we identify five suitable ones and reduce the number of suitable external stimuli from five to three with our optimization approach, especially with Algorithm 1 by increasing α. From the three non-zero external stimuli, we extract a tuple of two external stimuli which perform the desired switch.

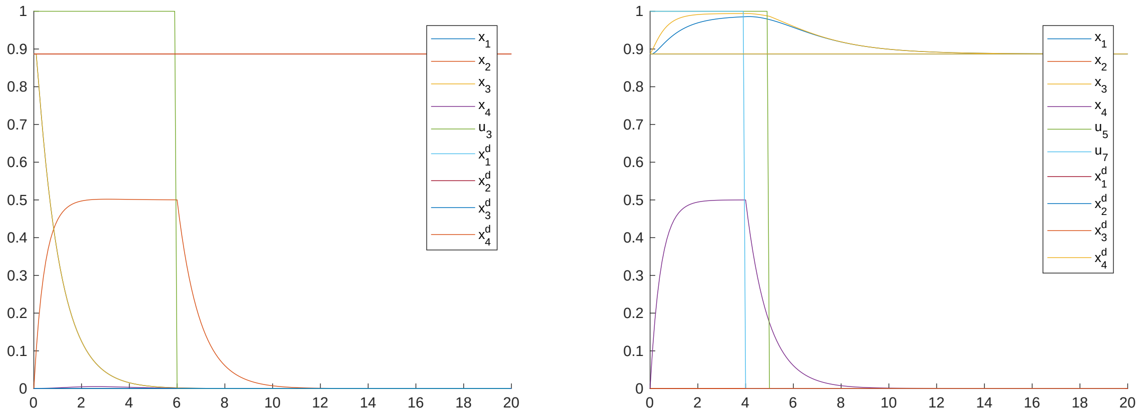

Figure H: The time is plotted on the abscissa and the activation level is plotted on the ordinate. These selections of applied external stimuli cannot perform the desired switch.

As well from Figure H, we tell that the selected external stimuli do not cover sufficient intervention points and do not affect the crucial network nodes to be able to perform the desired switch. That means that the set of controls has to interfere sufficiently many nodes from which our proposed framework then figures out the controls that perform the desired switch of steady states. Consequently, not any set of external stimuli can perform any desired switch. This observation can be interpreted as a filter of the network as only selected and rare situations where these coordinated external stimuli occur are able to induce changes of the cell behavior, for instance. As uncoordinated external stimuli do not switch the cell's behavior, the need for a coordinated occurrence of external stimuli to trigger the switch ensures that the cell is in the right place and right time to change its behavior assuming that this coordinated occurrence of external stimuli is unlikely somewhere else where it is not the right place and the right time.

Another example for too few applied external stimuli is (13) where just u_2 is applied to node 4, see Figure I. The switching of the external stimulus u_2 comes from the aim of Algorithm 1 of minimizing the target functional of (3). Through this switching of u_2, the activation level x_4 comes closer to the desired state which has less costs than letting u_2 be the constant zero function and the desired switch of steady states cannot be performed yet. If u_2 is switched off, then the activation level x_4 starts to decay immediately.

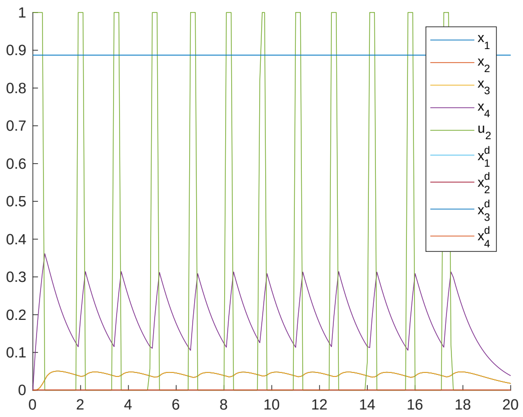

Figure I: The time is plotted on the abscissa and the activation level is plotted on the ordinate. The switching of u_2 minimizes the target functional in (3) and yet cannot perform a switch of the steady state.

On the other hand if we analyze (13) where once u_1, u_2 and u_3 are applied but u_3 to node 3 instead of node 2, see Figure Ja and once just u_1 and u_2, see Figure Jb, then there are more possible intervention points of external stimuli than needed for the desired switch in the first system. As a consequence, the switch of steady states is performed faster.

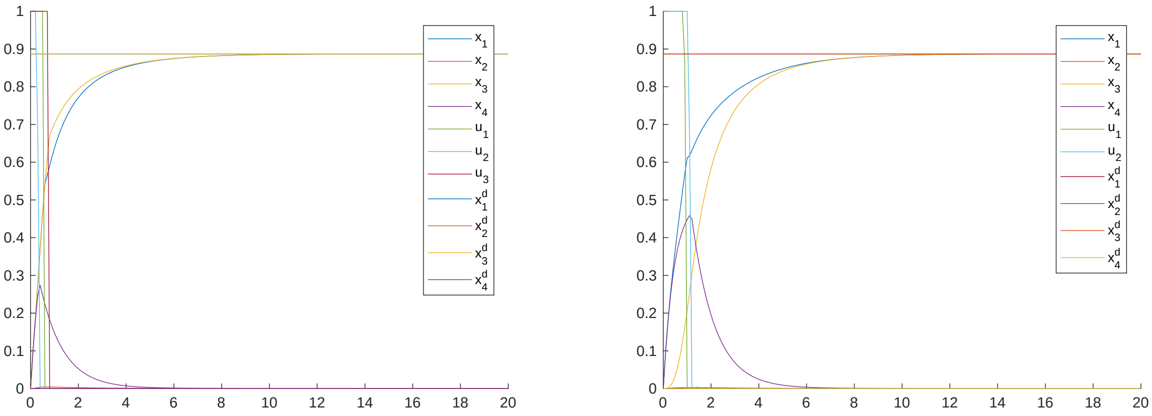

(a) Three external stimuli are applied. (b) Two external stimuli are applied

Figure J: The time is plotted on the abscissa and the activation level is plotted on the ordinate.

In our last experiment, we consider external stimuli which affect several nodes at once. This example is the blueprint how this framework can be combined with a data bank driven approach combining the information of an interactom and its affection by drugs. The information of the interactom is used for setting up the system of ordinary equations and all the possible drugs, which can also effect more than just one node, are represented by external stimuli that act on the nodes. Then by the calculations with our optimization framework, one can figure out the most effective drug combination.

We find a selection of external stimuli which steers our regulatory network from the steady state $x_0 = \begin{pmatrix} 0 & 0 & 0 & 0 \end{pmatrix}$ to the steady state $\begin{pmatrix} 0.8870 & 5.6662 \cdot 10^{-4} & 0.8870 & 5.6662 \cdot 10^{-4} \end{pmatrix}$, called Switch 1, then from the steady state $x_0 = \begin{pmatrix} 0.8870 & 5.6662 \cdot 10^{-4} & 0.8870 & 5.6662 \cdot 10^{-4} \end{pmatrix}$ to the steady state $\begin{pmatrix} 5.6662 \cdot 10^{-4} & 0.8870 & 5.6662 \cdot 10^{-4} & 0.8870 \end{pmatrix}$, called Switch 2 and finally from the steady state $x_0 = \begin{pmatrix} 5.6662 \cdot 10^{-4} & 0.8870 & 5.6662 \cdot 10^{-4} & 0.8870 \end{pmatrix}$ to the steady state $\begin{pmatrix} 0 & 0 & 0 & 0 \end{pmatrix}$ again, called Switch 3. We consider the following network

$$
\begin{aligned}
\frac{d}{dt}x_1 &= \frac{-\mathrm{e}^5 + \mathrm{e}^{-10\left(\frac{3}{2}\left(\frac{x_3+x_4}{1+x_3+x_4}\right)\left(1-\frac{11}{10}\frac{10x_2}{1+10x_2}\right)-0.5\right)}}{\left(1-\mathrm{e}^5\right)\left(1+\mathrm{e}^{-10\left(\frac{3}{2}\left(\frac{x_3+x_4}{1+x_3+x_4}\right)\left(1-\frac{11}{10}\frac{10x_2}{1+10x_2}\right)-0.5\right)}\right)} - x_1 + u_1\left(1-x_1\right) \\
\frac{d}{dt}x_2 &= \frac{-\mathrm{e}^5 + \mathrm{e}^{-10\left(\frac{3}{2}\left(\frac{x_3+x_4}{1+x_3+x_4}\right)\left(1-\frac{11}{10}\frac{10x_1}{1+10x_1}\right)-0.5\right)}}{\left(1-\mathrm{e}^5\right)\left(1+\mathrm{e}^{-10\left(\frac{3}{2}\left(\frac{x_3+x_4}{1+x_3+x_4}\right)\left(1-\frac{11}{10}\frac{10x_1}{1+10x_1}\right)-0.5\right)}\right)} - x_2 + u_3\left(1-x_2\right) \\
\frac{d}{dt}x_3 &= \frac{-\mathrm{e}^5 + \mathrm{e}^{-10\left(\frac{3}{2}\left(\frac{x_3+x_4}{1+x_3+x_4}\right)\left(1-\frac{11}{10}\frac{10x_2}{1+10x_2}\right)-0.5\right)}}{\left(1-\mathrm{e}^5\right)\left(1+\mathrm{e}^{-10\left(\frac{3}{2}\left(\frac{x_3+x_4}{1+x_3+x_4}\right)\left(1-\frac{11}{10}\frac{10x_2}{1+10x_2}\right)-0.5\right)}\right)} - x_3 + u_2\left(1-x_3\right) + u_1\left(1-x_3\right) - u_3x_3 \\
\frac{d}{dt}x_4 &= \frac{-\mathrm{e}^5 + \mathrm{e}^{-10\left(\frac{3}{2}\left(\frac{x_3+x_4}{1+x_3+x_4}\right)\left(1-\frac{11}{10}\frac{10x_1}{1+10x_1}\right)-0.5\right)}}{\left(1-\mathrm{e}^5\right)\left(1+\mathrm{e}^{-10\left(\frac{3}{2}\left(\frac{x_3+x_4}{1+x_3+x_4}\right)\left(1-\frac{11}{10}\frac{10x_1}{1+10x_1}\right)-0.5\right)}\right)} - x_4 + u_2\left(1-x_4\right) - u_1x_4
\end{aligned} \tag{15}
$$

where u_1 activates node 1 and node 3 and inhibits node 4, u_2 activates node 3, u_3 activates node 2 and inhibits node 3. The results from Algorithm 1 with $\alpha = 0.1$, $T = 20$, $dt = 0.1$, $\epsilon = 10^{-6}$ and ${}^0u$ with constant zero values for Switch 1 can be seen in Figure K, for Switch 2 in Figure L where the oscillations of u_2 and u_3 is not necessary for the switch, see Figure M and finally, for Switch 3, see Figure N.

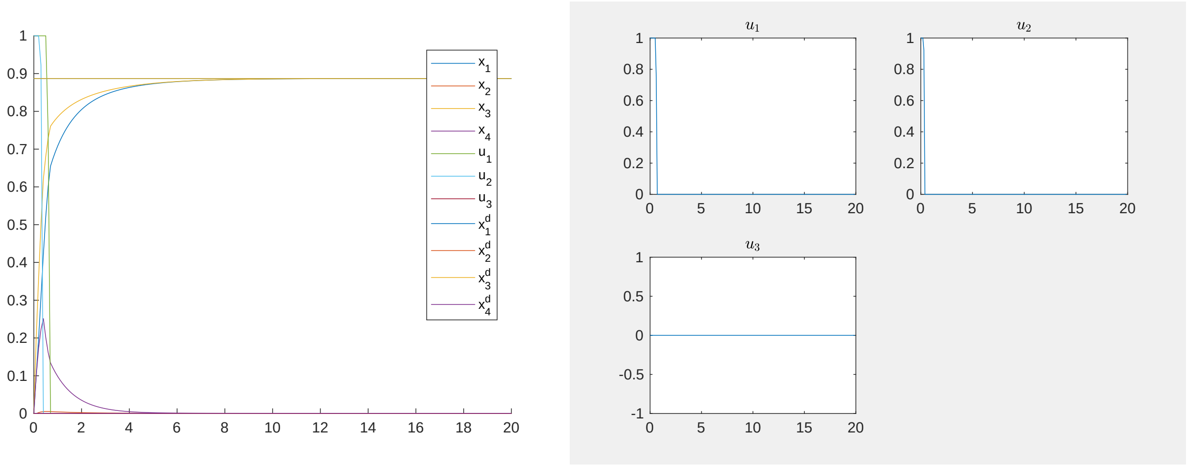

Figure K: Switch 1. The time is plotted on the abscissa and the activation level is plotted on the ordinate.

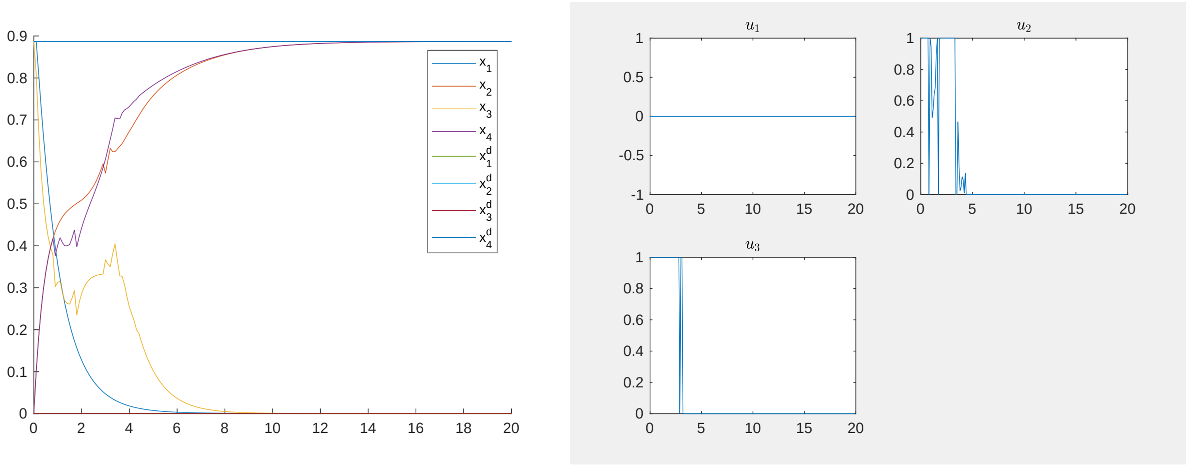

Figure L: Switch 2. The time is plotted on the abscissa and the activation level is plotted on the ordinate.

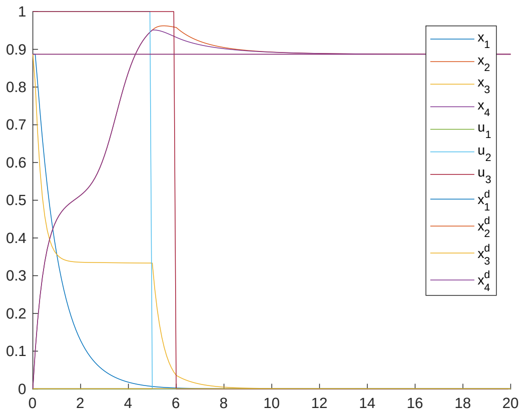

Figure M: The time is plotted on the abscissa and the activation level is plotted on the ordinate. Applying u_2 and u_3 in this way performs the desired Switch 2 as well.

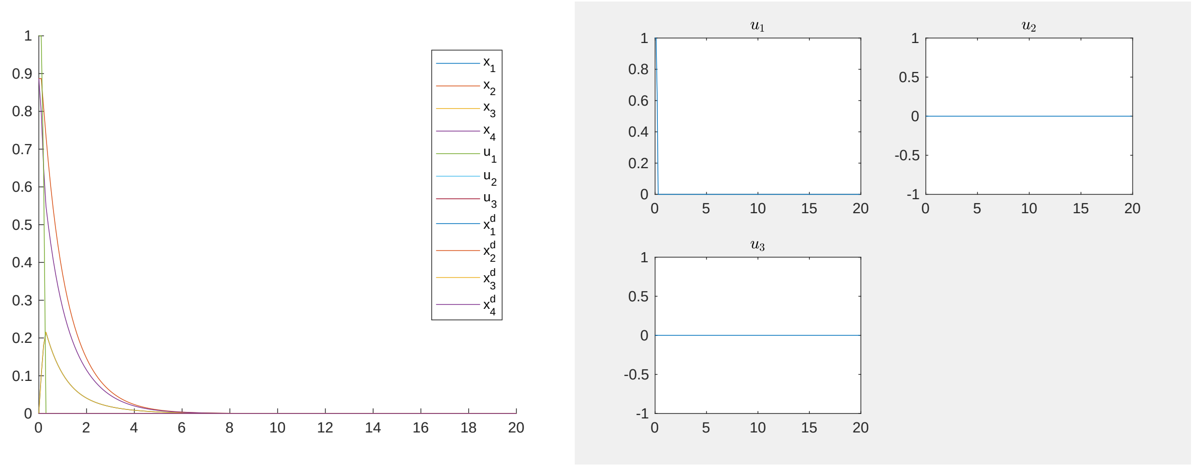

Figure N: Switch 3. The time is plotted on the abscissa and the activation level is plotted on the ordinate.

3 A short introduction to the software package Jimena2

In this section, we give a short introduction into the software package provided with this work that extends the mentioned Jimena [9]. First, we have the Matlab framework. It consists of the following functions. The main.m is the central function which has to be started with Matlab for the computations. The state (2) is solved with the function forward.m. The desired steady state x_d is set with the function get_xd.m. The function combinatorial_method.m is the implementation of Algorithm 3. Algorithm 1 is implemented in projected_gradient_method.m and Algorithm 2 is implemented in SQH_method.m. For the different algorithms, we have the following auxiliary functions, these are createJacobian.m, creating the Jacobian of f with respect to x and u, projection.m, projecting each component of u into $[0,1]$, setControls.m, setting the value of the external stimuli to 1 for a certain period of time, else zero, the function backward.m or backward_SQH.m, solving the adjoint equation (7) or (10), respectively, for given state x and external stimuli u, the function get_J.m or get_J_SQH.m, respectively, calculating the value of the target functional J of (1), get_gradient.m, assembling the gradient (5) for the projected gradient method, drawStimuli.m, drawing the curves of the resulting external stimuli and drawStates.m, drawing the corresponding states. In order to do the calculation above, one needs a Matlab version with a symbolic math toolbox. Additionally the parallel computing toolbox is recommended. If this toolbox is not available, then just put “for” instead of “parfor” in the function createJacobian.m. In order to do the calculation, one has to execute the main.m with Matlab while all other functions mentioned above are in the same folder as the main.m.

Especially if the network consists of many nodes and thus the corresponding system of ordinary differential equations is large, it is convenient to create the main.m, including the corresponding right hand-side of the system of ordinary differential equations f, automatically by a program. For this purpose, we provide a tool implemented with Java. Firstly, we create our network with the yEd Graph editor from yWorks as follows. An activating connection between two nodes is created with a “standard” target arrow, see Figure O left hand-side and an inhibiting connection between two nodes is created with a “t_shape” target arrow, see Figure O right hand-side.

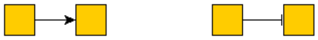

Figure O: An activating connection between two nodes left hand-side and an inhibiting connection between two nodes right hand-side.

Secondly, one runs the java application jimena2.jar and imports the network file under “Network”. Then one analyzes the network with “Find stable states” under “Analysis”. Clicking “Switch Analyzer” opens a window where the parameters of the optimization framework can be set including the following. One can select initial and target state, that means the desired state x_d and add activating and inhibiting external stimuli to nodes by “Positive regulation” or “Negative regulation”, respectively. The “Interval” corresponds to the step size dt and “Horizon” to the time horizon T. It is recommendable to take the same numbers as used for finding the stable states. The parameters “Alpha”, “Beta”, “Gamma”, “H” correspond to the model parameters of Equation (1) in the main manuscript where we assume that the parameters do not depend on the node but have the same value for all nodes to keep the entry clear. The parameter

"AlphaW" corresponds to the weight α in J of (1). By clicking "Generate" one creates a Matlab file which can have any name, we denote it with main.m, but has to be saved within the folder where the other necessary functions mentioned above are saved. Then, one can again execute this generated file with Matlab.

The main.m file outputs the number of external stimuli in the Matlab console which are non zero and gives feedback if the switch is performed with these external stimuli, besides other useful information regarding the methods used to calculate the external stimuli. However, the most import information from the console is what external stimuli are different from the constant zero function because these are the stimuli of interest for the desired switch. Additionally, the time curve of the nodes and of the external stimuli are saved in the text file "x.txt" and "u.txt", respectively. With the functions drawStates.m or drawStimuli.m one can look at the time curves. For this purpose, one has to insert a vector of numbers of the nodes or external stimuli, respectively, one would like to see, the name of the corresponding file, which is standard "x.txt" and "u.txt", respectively, but can have any name if the files of interest have been renamed, and how many columns of figures are supposed to be in the plot. For example one can enter in the Matlab console drawStimuli([1,2,5],'u.txt',2) to see external Stimulus 2,3 and 5 where at most two figures are in a row.

At the beginning of the main.m function, one can change parameters important for the Matlab framework. We have "tol1" which corresponds to the tolerance which a node's activation level is allowed to differ at most from the desired value to decide that this node has reached its desired state, "tol2" is the stopping criterion of the sequential quadratic Hamiltonian method, "tol3" is the the stopping criterion for the projected gradient method, "T_int" is a tolerance used in the combinatorial method to determine the smallest period of application of external stimuli still causing a desired switch, "max_Num" is the maximum number of different external stimuli applied at once for inducing a desired switch in the combinatorial method. The flag "combi_method" is set to 1 if a combinatorial search of external stimuli is supposed to be performed before a local optimization method and 0 if not. If "local_optimization_method" equals zero, then no local optimization method is performed, while if it equals 1, then the sequential quadratic Hamiltonian method is performed and if it equals 2, then the projected gradient method is performed. The standard value is 1. In the struct OCP, the element "numNodes" corresponds to the number of nodes of the network and "numControls" to the number of external stimuli. The element "timeInterval" is the step size in which the time is discretized, "timeHorizon" is the final time and duration of the simulation as the simulation is assumed to start at $t = 0$. The element "alpha" corresponds to the "AlphaW" in the switch analyzer and in the element "initialState" the initial state of the network is denoted. All the elements of OCP are set accordingly by the switch analyzer if used to create the Matlab main.m file.

A further installation tutorial is available at https://www.biozentrum.uni-wuerzburg.de/bioinfo/computing/jimena2/.

References

[1] Herbert Amann and Joachim Escher. *Analysis II*. Birkhäuser, 2008.

[2] Dimitri P. Bertsekas. *Nonlinear programming*. Athena scientific Belmont, 1999.

[3] Alfio Borzì and Volker Schulz. *Computational optimization of systems governed by partial differential equations*. SIAM, 2011.

[4] Alexandra Brietz, Kristin V. Schuch, Gaby Wangorsch, Kristina Lorenz, and Thomas Dandekar. Analyzing ERK 1/2 signalling and targets. *Molecular bioSystems*, 12(8):2436–2446, 2016.

[5] John C. Butcher. *Numerical methods for ordinary differential equations*. John Wiley & Sons, 2016.

[6] Claudia Goettlich, Lena C. Mueller, Meik Kunz, Franziska Schmitt, Heike Walles, Thorsten Walles, Thomas Dandekar, Gudrun Dandekar, and Sarah L. Nietzer. A combined 3D tissue engineered in vitro/in silico lung tumor model for predicting drug effectiveness in specific mutational backgrounds. *Journal of visualized experiments: JoVE*, (110):e53885–e53885, 2016.

[7] Aleksandr D. Ioffe and Vladimir M. Tihomirov. *Theory of extremal problems*, volume 6. Elsevier, 2009.

[8] Kazufumi Ito and Karl Kunisch. *Lagrange multiplier approach to variational problems and applications*. SIAM, 2008.

[9] Stefan Karl and Thomas Dandekar. Jimena: efficient computing and system state identification for genetic regulatory networks. *BMC bioinformatics*, 14(1):306, 2013.

[10] Luis Mendoza and Ioannis Xenarios. A method for the generation of standardized qualitative dynamical systems of regulatory networks. *Theoretical Biology and Medical Modelling*, 3(1):13, 2006.

[11] Christina Pachel, Denise Mathes, Barbara Bayer, Charlotte Dienesch, Gaby Wangorsch, Wolfram Heitzmann, Isabell Lang, Hossein Ardehali, Georg Ertl, Thomas Dandekar, et al. Exogenous administration of a recombinant variant of TWEAK impairs healing after myocardial infarction by aggravation of inflammation. *PLoS One*, 8(11):e78938, 2013.

[12] Anna T. Stratmann, David Fecher, Gaby Wangorsch, Claudia Göttlich, Thorsten Walles, Heike Walles, Thomas Dandekar, Gudrun Dandekar, and Sarah L. Nietzer. Establishment of a human 3D lung cancer model based on a biological tissue matrix combined with a boolean in silico model. *Molecular oncology*, 8(2):351–365, 2014.
